# Japanese Society for Cancer of the Colon and Rectum (JSCCR) guidelines 2024 for the treatment of colorectal cancer

**DOI:** 10.1007/s10147-025-02899-8

**Published:** 2025-11-04

**Authors:** Yusuke Kinugasa, Kay Uehara, Kensei Yamaguchi, Yutaka Saito, Keiko Murofushi, Tamotsu Sugai, Megumi Ishiguro, Soichiro Ishihara, Hideki Ueno, Shiro Oka, Takeshi Kato, Yukihide Kanemitsu, Hisato Kawakami, Hirotoshi Kobayashi, Yoshihiro Sakamoto, Manabu Shiozawa, Akio Shiomi, Eiji Shinozaki, Hirotoshi Takiyama, Hiroya Taniguchi, Takako Eguchi Nakajima, Kinichi Hotta, Keiji Matsuda, Kohei Murata, Satoshi Morita, Kentaro Yamazaki, Masahiro Yoshida, Naohiko Yamaguchi, Hiroyasu Kagawa, Shinichi Yamauchi, Yoichi Ajioka, Japanese Society for Cancer of the Colon and Rectum

**Affiliations:** 1 Department of Gastrointestinal Surgery, Institute of Science Tokyo (Science Tokyo) Graduate School, 1-5-45 Yushima, Bunkyo-ku, Tokyo, 113-8519 Japan; 2 Department of Gastroenterological Surgery, Nippon Medical School, Tokyo, Japan; 3 Department of Gastroenterological Chemotherapy, Cancer Institute Hospital of the Japanese Foundation for Cancer Research, Tokyo, Japan; 4 Endoscopy Division, National Cancer Center Hospital, Tokyo, Japan; 5 Division of Radiation Oncology, Department of Radiology, Tokyo Metropolitan Cancer and Infectious Diseases Center Komagome Hospital, Tokyo, Japan; 6 Pathology, Southern Tohoku Research Institute for Neuroscience Southern Tohoku General Hospital, Fukushima, Japan; 7 Health Science Research and Development Center, Institute of Science Tokyo Hospital, Tokyo, Japan; 8 Department of Surgical Oncology, Graduate School of Medicine, The University of Tokyo, Tokyo, Japan; 9 Department of Surgery, National Defense Medical College, Saitama, Japan; 10 Department of Gastroenterology, Graduate School of Biomedical and Health Sciences, Hiroshima University, Hiroshima, Japan; 11 Department of Surgery, NHO Osaka National Hospital, Osaka, Japan; 12 Department of Colorectal Surgery, National Cancer Center Hospital, Tokyo, Japan; 13 Department of Medical Oncology, Tohoku University Hospital, Miyagi, Japan; 14 Department of Surgery, Mizonokuchi Hospital, Teikyo University School of Medicine, Kanagawa, Japan; 15 Department of Gastroenterological and General Surgery, Kyorin University School of Medicine, Tokyo, Japan; 16 Department of Gastrointestinal Surgery, Kanagawa Cancer Center, Kanagawa, Japan; 17 Division of Colon and Rectal Surgery, Shizuoka Cancer Center, Shizuoka, Japan; 18 QST Hospital, National Institutes for Quantum Science and Technology, Chiba, Japan; 19 Department of Clinical Oncology, Aichi Cancer Center Hospital, Nagoya, Japan; 20 Department of Early Clinical Development, Kyoto University Graduate School of Medicine, Kyoto, Japan; 21 Division of Endoscopy, Shizuoka Cancer Center, Shizuoka, Japan; 22 Department of Surgery, Fraternity Memorial Hospital, Tokyo, Japan; 23 Department of Surgery, Osaka Rosai Hospital, Osaka, Japan; 24 Department of Biomedical Statistics and Bioinformatics, Graduate School of Medicine, Kyoto University, Kyoto, Japan; 25 Division of Gastrointestinal Oncology, Shizuoka Cancer Center, Shizuoka, Japan; 26 Department of Hepato-Biliary-Pancreatic and Gastrointestinal Surgery, School of Medicine, International University of Health and Welfare, Chiba, Japan; 27 Library of SEIREI, SAKURA Citizen Hospital, Sakura, Japan; 28 Division of Molecular and Diagnostic Pathology, Graduate School of Medical and Dental Sciences, Niigata University, Niigata, Japan

**Keywords:** Colorectal cancer, Guideline, Surgery, Chemotherapy, Endoscopy, Radiotherapy

## Abstract

The number of deaths from colorectal cancer in Japan continues to rise, with over 50,000 deaths recorded in 2018. In the 2024 edition, revisions to all aspects of treatment were undertaken, with corrections and additions made based on knowledge gained since the 2022 version (drug therapy) and the 2019 version (other treatments). The Japanese Society for Cancer of the Colon and Rectum (JSCCR) guidelines 2024 for the treatment of colorectal cancer have been prepared to present standard treatment strategies, reduce disparities among institutions, avoid both unnecessary and insufficient treatment, and enhance mutual understanding between healthcare professionals and patients by making these guidelines accessible to the public. These guidelines were developed through consensus by the JSCCR Guideline Committee, following a careful review of evidence retrieved from literature searches and considering the medical insurance system and actual clinical practice in Japan. Therefore, these guidelines serve as a tool for managing colorectal cancer in real-world clinical settings. More specifically, they can be used to support obtaining informed consent from patients and selecting the most appropriate treatment method for each patient. Controversial topics were selected as clinical questions, and recommendations were provided. Each recommendation is accompanied by an evidence classification and a recommendation category, both based on consensus reached by the Guideline Committee members. This article presents the English version of the JSCCR guidelines 2024.

## Introduction

1. Guideline objectives

According to the Ministry of Health, Labor and Welfare’s Summary of Population-Based Cancer Registry, there were 158,000 colorectal cancer cases in Japan in 2016, making it the most prevalent cancer type. Additionally, Vital Statistics data indicate that deaths from colorectal cancer exceeded 50,000 in 2018, ranking it as the second most common cause of cancer-related mortality. In light of this situation, improving treatment outcomes for colorectal cancer has become an urgent national priority. The Japanese Society for Cancer of the Colon and Rectum (JSCCR) guidelines 2024 for the treatment of colorectal cancer (hereinafter “these Guidelines”) were developed for physicians (general practitioners and specialists) who treat individuals with colorectal cancer at various stages and under diverse clinical conditions, with the following objectives [1].

1. to show standard treatment strategies for colorectal cancer
2. to eliminate disparities among institutions in terms of treatment
3. to eliminate unnecessary treatment and insufficient treatment
4. to deepen mutual understanding between healthcare professionals and patients by making these guidelines available to the general public.

The expected benefits of creating these guidelines are

(1) improvement of the treatment of colorectal cancer in Japan; (2) improvement of the results of treatment; (3) reduction of the human and financial burden; and (4) increased benefits for patients.

2. How to use these guidelines

These guidelines were developed based on the consensus of the JSCCR, which respects the evidence obtained from literature reviews and considers the Japanese medical insurance system and the actual conditions in clinical practice. They can be used as a tool when implementing colorectal cancer treatment in clinical practice. Specifically, they support formulating treatment plans for individual cases and assist in obtaining informed consent from patients. However, these guidelines are intended to provide direction for formulating colorectal cancer treatment plans and do not regulate plans or methods beyond those described. They can also serve as documentation to explain the rationale for selecting treatment policies or methods that differ from those outlined. In such cases, it is essential to provide sufficient explanations to the patient and their family, obtain consent, and ensure that the approach is logical and ethical to be accepted by third parties.

The JSCCR assumes responsibility for the content of these Guidelines; however, responsibility for individual treatment outcomes rests with the treating clinician, and neither the JSCCR nor the Guidelines Committee bears responsibility.

3. User

The intended users of these guidelines are primarily clinicians involved in the treatment of colorectal cancer.

4. How to develop these guidelines

Recording methods

The concept of the first edition was retained, presenting a treatment algorithm, providing a brief explanation of it, and adding comments on matters requiring further clarification. Since the 2009 edition, controversial issues have been raised as clinical questions (CQs) through consensus of the guideline creation committee, using a format in which recommendations are written. The 2019 edition retained this format, revising and adding CQs in pharmacotherapy based on findings since the 2016 edition and in other areas since the 2014 edition. In the 2022 edition, while retaining the format, the CQ wording was clarified and made unambiguous, and when comparing multiple interventions, the wording was made flexible to be useful in clinical practice rather than insisting on ranking all options.

In the 2024 edition, a statement of the agreement rate was added to make any differences of opinion among committee members transparent. With regard to the revision of CQs, there has been an addition, modification, or deletion of CQs based on new treatments in actual clinical practice and new evidence, with a focus on issues that are clinically important at this time.

In explaining the CQs, emphasis was placed on keeping explanations clear and of balanced length. When citing a large number of clinical trials, descriptions of specific numerical values and other research details were simplified.

Clinicopathological terms followed those in the Japanese Classification of Colorectal, Appendiceal, and Anal Carcinoma, third English edition [2].

5. Evidence level/strength of recommendations of CQs

The recommendation statements for CQs are accompanied by the level of evidence and the strength of recommendation, determined through the following process.

Evidence level

We comprehensively collected papers related to the CQs and categorized the evidence for each important outcome by study design [3]. We then evaluated the quality of the literature and the overall body of evidence using the GRADE* system [4–22] (Table 1) and, based on this assessment, determined the level of evidence for the CQs (Table 2).

**Table 1:** Rating the quality of evidence

Step 1 (evaluation of individual study): study design, evaluation of bias risk, create structured abstract	
Step 2 (overall rating for each important outcome across studies);	
1. Initial quality of a body of evidence: evaluation of each study design group	
Systematic reviews, meta-analysis, randomized-controlled trials = “initial quality A (high level)”	
Observation studies, cohort studies, case control studies = “initial quality C (low level)	
Case series, case reports = “initial quality D (very low level)	
2. Five reasons to possibility rate down the quality	
Risk of bias	
Inconsistency in results	
Indirectness of evidence	
Data imprecision	
High possibility of publication bias	
3. Three reasons to possibility rate up the quality	
Large effect with no confounding factors	
Dose–response gradient	
Possible confounding factors are weaker than actual effects	
4. We evaluate 1- > 2- > 3, and assess the quality of a body of evidence

**Table 2:** Definition of levels of evidence (Ref. [13])

A (high)	We are very confident in the effect estimate
B (moderate)	We are moderately confident in the effect estimate: the true effect is likely to be close to the estimate of the effect, but there is a possibility that it is substantially different
C (low)	Our confidence in the effect estimate is limited: the true effect may be substantially different from the estimate of the effect
D (very low)	We have very little confidence in the effect estimate: the true effect is likely to be substantially different from the estimate of effect

Strength of recommendation

Based on the outcomes and evidence levels obtained through the above process, a recommendation draft was prepared. The guideline development committee members evaluated this draft at a consensus conference and determined the strength of the recommendation (Table 3). In the CQ text, recommendations were expressed directly, and varied expressions were removed.

**Table 3:** Strength of recommendation (Ref. [23])

Strength of recommendation	
1 (Strong recommendation)	Strong “For” an intervention
	Strong “Against” an intervention
2 (Weak recommendation)	Weak “For” an intervention
	Weak “Against” an intervention

The strength of the recommendation was determined by evaluating the recommendation statement according to four criteria: ① quality of evidence, ② patients’ views and preferences, ③ benefits and harms, and ④ cost-effectiveness, and was determined based on a vote according to the GRADE grid method [10].

## Methods

1. There are five voting options:

① Strong “For” intervention

② A Weak “For” intervention

③ Weak “Against” intervention

④ Strong “Against” intervention

⑤ Not graded.

2. With one vote, if 70% or more of the votes were obtained in any of ① to ⑤, it was considered a final decision.

If this criterion cannot be met, then the following shall be applied:

- If ① + ② exceeds 50%, ③ + ④ is 20% or lower, “weakly recommend to perform.”
- If ③ + ④ exceeds 50%, ① + ② is 20% or lower, “weakly recommend not to perform.”

3. If none of the conditions listed in 2 were met in the first vote, it would be deemed that "no consensus was reached," and the results of the vote would be disclosed, and renegotiations would be held taking into account Japan's medical situation, followed by a revote.

If no consensus was reached after the second vote, the recommendation was deemed “no particular recommendation.”

4. Literature search

The literature search was conducted by a medical librarian, using PubMed and Ichushi-Web (https://search.jamas.or.jp/). A search formula was developed in consultation with the committee members responsible for each item, and English and Japanese literature was extracted from both databases.

For CQs continued from the previous edition, literature from February 2021 to December 2022 was searched to identify the latest studies to supplement the adopted literature from the previous edition. For new CQs, each committee member determined an appropriate starting point for the search period, and searches were conducted through December 2022, similar to continuing CQs. All searches were performed in early January 2023. The total number of additional searches was 10,494, and the total number of additional selections was 3,524.

In addition, each committee member conducted a manual search for literature not retrieved by the search formula but deemed necessary for creating each item.

The results of the literature search and selection for the 2022 edition and the current edition are shown in Table 4. Of the 28,356 articles identified (19,178 from PubMed, 9,178 from Ichushi, and 751 from manual searches), 7,376 were selected. For this edition, 3,652 new documents were obtained and thoroughly reviewed.

**Table 4:** Number of scientific articles retrieved and selected

	Retrieved		Selected		Retrieved manually
	PubMed	Ichushi	PubMed	Ichushi	
(1) Endoscopic treatment	2208	685	172	74	93
(2) Surgical treatment	7825	3852	2148	782	146
(3) Radiotherapy	1331	193	377	28	72
(4) Systematic therapy	5586	2794	1915	474	370
(5) Others	2285	1654	445	210	70
Total	19,178	9178	5057	1568	751

**Fig. 1:**
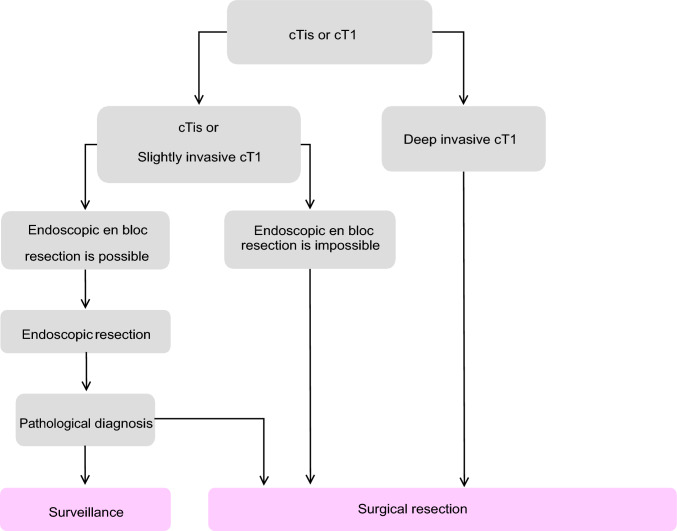
Treatment strategies for cTis and cT1 colorectal cancer

**Fig. 2:**
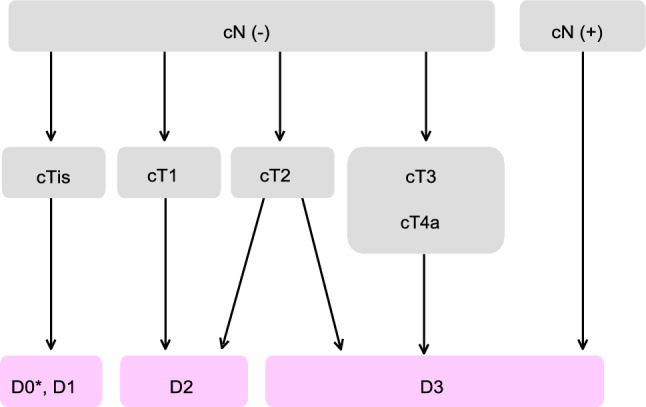
Surgical treatment strategies for cStage 0 to cStage III colorectal cancer

**Fig. 3:**
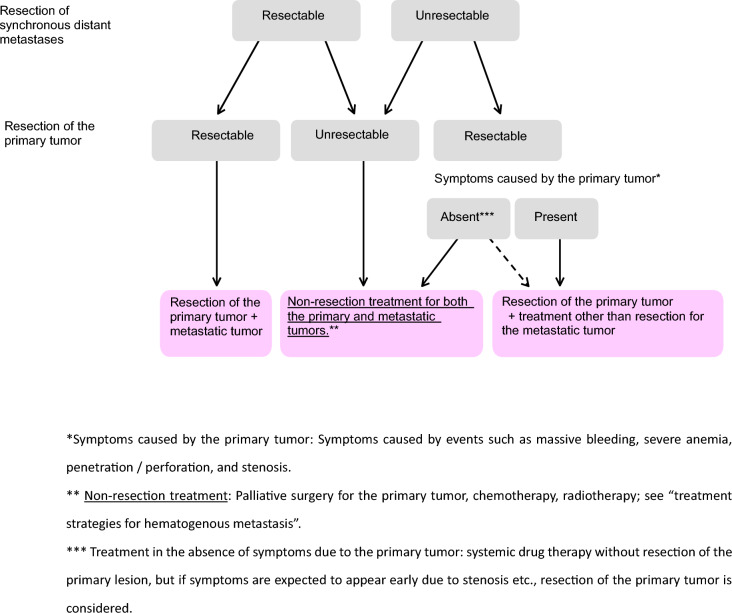
Treatment strategies for Stage IV colorectal cancer

**Fig. 4:**
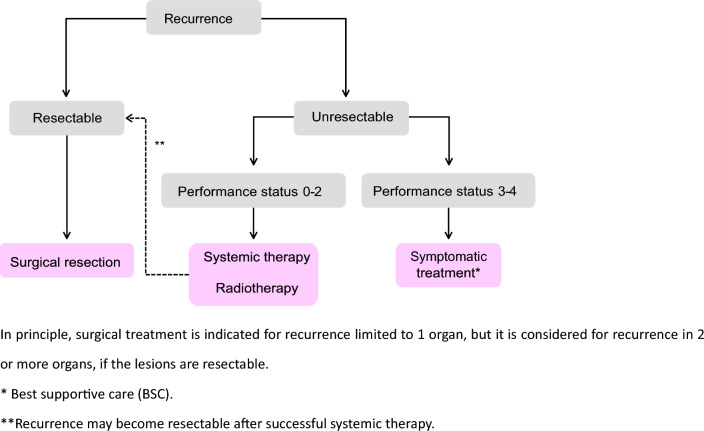
Treatment strategies for recurrent colorectal cancer

**Fig. 5:**
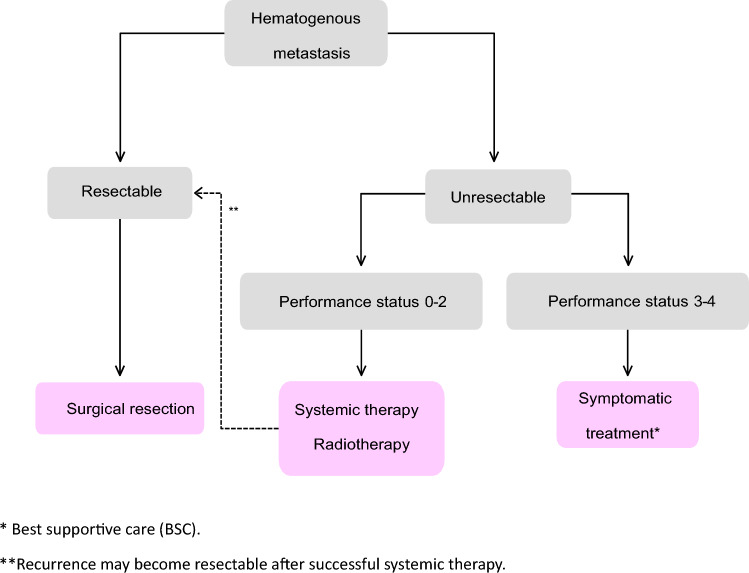
Treatment strategies for hematogenous metastases

**Fig. 6:**
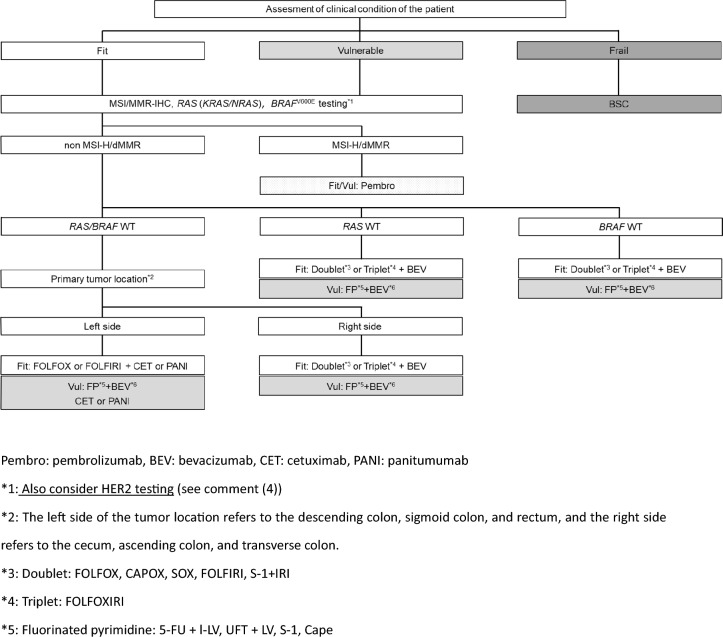
Steps in the decision‑making process for the first‑line treatment in unresectable colorectal cancer

**Fig. 7:**
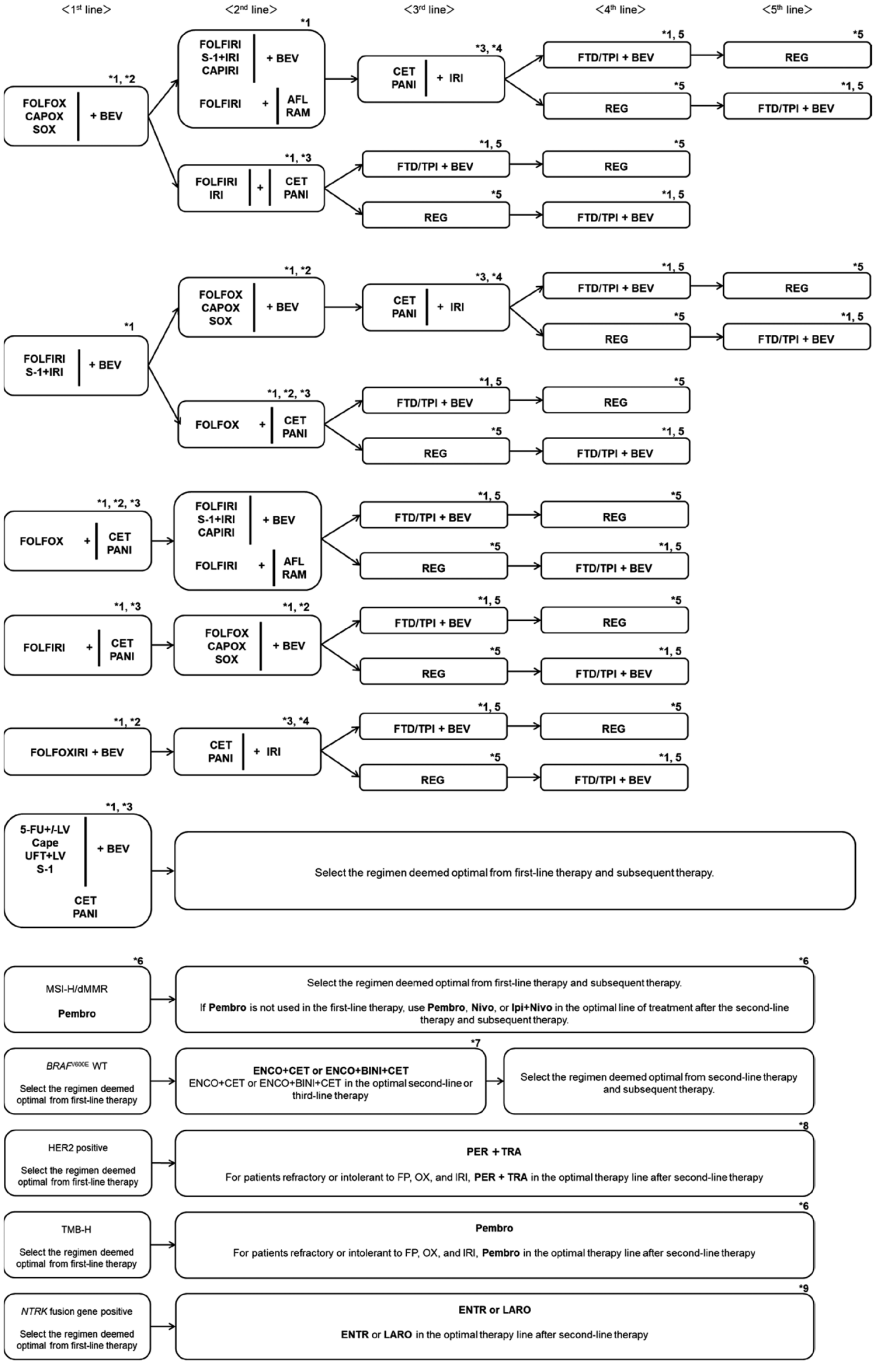

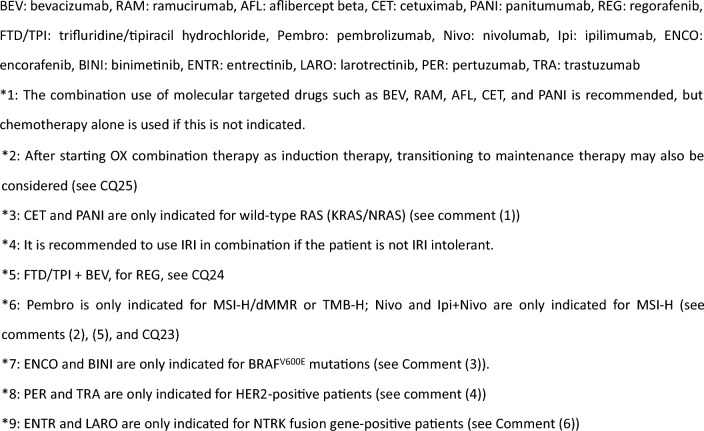
Systemic therapy algorithm for unresectable colorectal cancer

**Fig. 8:**
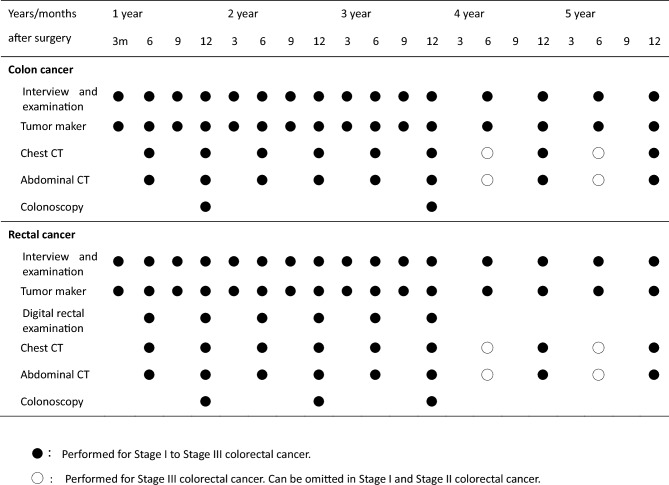
An example of a surveillance schedule after curative resection of pStage I to pStage III colorectal cancer

**Fig. 9:**
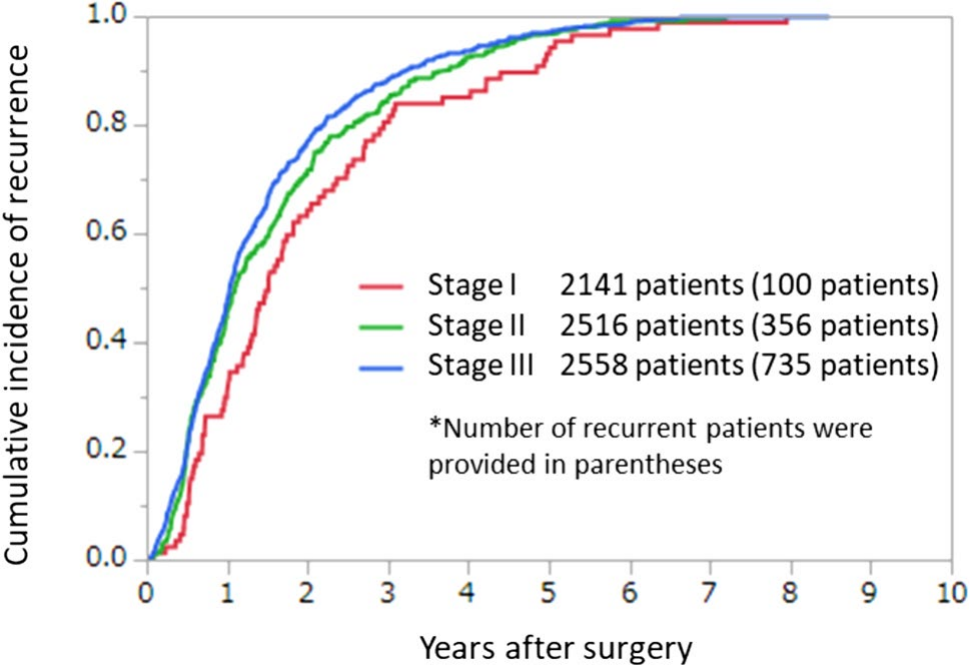
Cumulative incidence of recurrence according to stage (JSCCR colorectal cancer registry: patients in the year 2014)

**Fig. 10:**
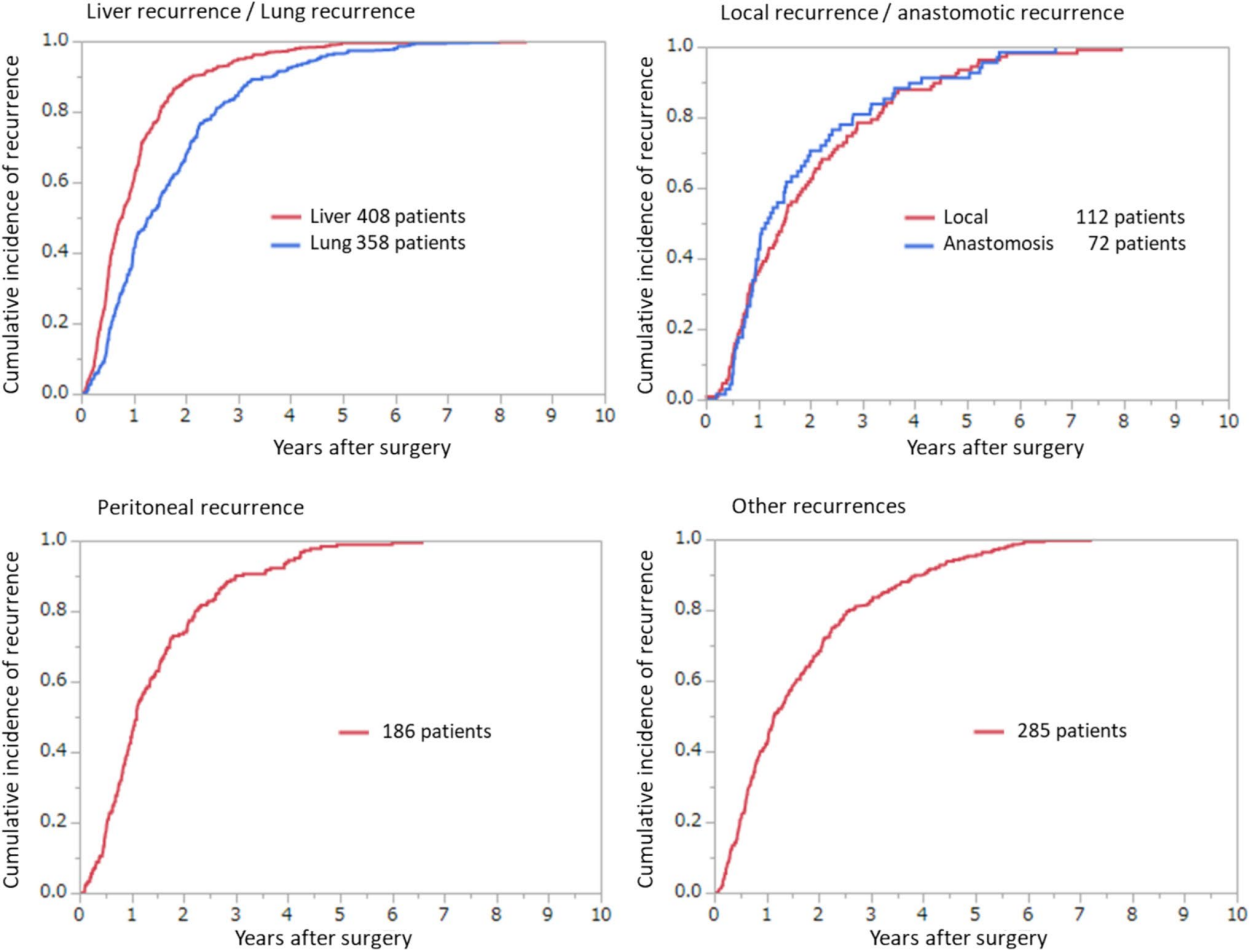
Cumulative incidence of recurrence according to the site of recurrence (JSCCR colorectal cancer registry: patients in the year 2014)

**Fig. 11:**
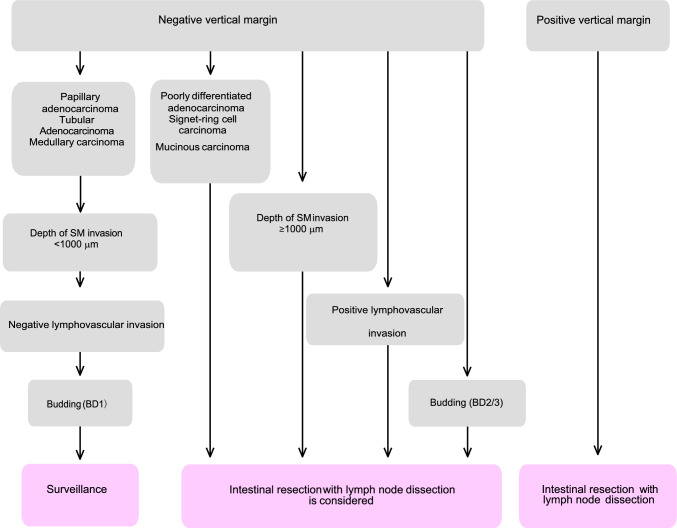
Treatment strategies for pT1 cancer after endoscopic resection

**Table 5:** Incidences of lymph-node metastasis according to tumor location and depth of tumor invasion

	No. of patients	Extent of lymph-node metastasis detected histologically					
		N0 (%)	N1a (%)	N1b (%)	N2a (%)	N2b (%)	N3 (%)
All sites							
T1	5314	89.6	6.9	2.6	0.4	0.2	0.3
T2	4522	78.8	10.3	6.6	2.3	0.8	1.2
T3	12,838	61.3	14.1	12.3	5.6	3.1	3.6
T4a	3349	45.0	15.7	17.1	6.8	9.5	5.8
T4b	1311	57.7	13.2	11.5	4.7	6.1	6.8
Colon							
T1	3206	90.4	6.6	2.4	0.3	0.06	0.2
T2	2179	80.7	10.5	5.9	2.1	0.4	0.4
T3	7648	65.5	14.4	11.2	5.1	1.7	2.2
T4a	2466	47.2	15.7	16.7	5.9	8.4	6.1
T4b	820	60.2	15.4	11.3	3.3	5.6	4.2
Rectosigmoid							
T1	583	88.7	8.4	2.1	0.5	0.2	0.2
T2	546	76.6	11.4	8.2	2.6	1.1	0.2
T3	1575	59.2	14.7	14.3	6.1	3.9	1.8
T4a	491	39.3	16.5	20.0	8.8	10.8	4.9
T4b	155	63.2	7.7	8.4	9.7	6.5	4.5
Upper rectum							
T1	520	88.1	8.1	2.9	0.8	0.2	0.0
T2	612	77.5	9.8	7.5	3.1	1.3	0.8
T3	1641	54.1	14.6	15.7	7.8	5.0	2.9
T4a	345	37.7	15.4	17.1	11.0	14.8	4.1
T4b	96	54.2	10.4	14.6	8.3	7.3	5.2
Lower rectum							
T1	970	88.4	6.7	3.3	0.4	0.4	0.8
T2	1126	76.6	10.0	6.8	2.1	1.2	3.2
T3	1903	52.4	12.5	11.9	5.8	6.5	11.0
T4a	45	44.4	11.1	13.3	4.4	15.6	11.1
T4b	191	45.0	11.5	13.6	4.7	7.9	17.3

JSCCR colorectal cancer registry: patients in years 2008–2013. Depth of invasion and the degree of lymph-node metastasis were determined according to the rules set forth in the “Japanese Classification of Colorectal Carcinoma” (9th edition)

**Table 6:** Lateral dissection and lateral metastasis of rectal cancer

No. of patients		No. of patients who underwent lateral dissection	Lateral dissection rate (%)	No. of patients with lateral metastasis	Lateral metastasis-rate (percentage of all patients) (%)	Lateral metastasis rate (percentage of patients who underwent lateral dissection) (%)
RS						
T1	124	0	0	0	0.0	0.0
T2	127	6	4.7	0	0.0	0.0
T3	316	24	7.5	0	0.0	0.0
T4a	177	8	4.5	0	0.0	0.0
T4b	32	14	43.8	1	3.1	7.1
Total	776	52	6.7	1	0.1	1.9
Ra						
T1	138	5	3.6	0	0.0	0.0
T2	149	18	12.1	0	0.0	0.0
T3	230	58	25.2	4	1.7	6.9
T4a	181	59	32.6	7	3.9	11.9
T4b	15	8	53.3	0	0.0	0.0
Total	713	148	20.8	11	1.5	7.4
Rb						
T1	234	37	15.8	2	0.9	5.4
T2	372	218	58.6	20	5.4	9.2
T3	350	230	65.7	28	7.7	12.2
T4a	412	319	77.4	75	18.0	23.5
T4b	59	48	81.4	17	28.8	35.4
Total	1427	852	59.7	142	9.8	16.7

Project study by the JSCCR: patients in years 1991–1998

*RS* rectosigmoid, *Ra* upper rectum, *Rb* lower rectum

**Table 7:** Curative resection rate according to pStage (lower rows: no. of patients)

pStage	I	IIa	IIb	IIc	IIIa	IIIb	IIIc	I–III
All site	99.7%	98.6%	95.2%	87.1%	99.5%	96.6%	89.7%	97.4%
	8128	7653	1471	738	1256	5266	2122	26,624
Colon	99.8%	99.1%	95.7%	89.6%	99.5%	96.7%	92.1%	97.8%
	4558	4881	1137	480	635	3130	1103	15,924
Rectosigmoid	99.9%	99.2%	95.7%	59.7%	100.0%	97.7%	93.6%	98.2%
	911	909	185	97	166	724	264	3256
Upper rectum	99.8%	98.3%	90.6%	74.5%	99.4%	96.7%	89.6%	96.9%
	907	856	128	51	166	749	268	3125
Lower rectum	99.3%	96.0%	94.7%	78.6%	99.3%	95.2%	82.8%	95.7%
	1673	967	19	84	281	644	443	4111
Anal canal	0.0%	0.0%	100.0%	94.6%	100.0%	89.5%	70.6%	88.5%
	0	0	2	26	8	19	34	208

JSCCR colorectal cancer registry: patients in years 2008–2013. Curative resection rate = number of patients with histological curability A cancer/total number of patients who underwent surgery. Depth of invasion and the degree of lymph-node metastasis were determined according to the rules set forth in the “Japanese Classification of Colorectal Carcinoma” (9th edition)

**Table 8:** Cumulative 5‑year survival rate according to tumor location (lower rows: no. of patients)

Stage	I	IIa	IIb	IIc	IIIa	IIIb	IIIc	I–III	IV (CurB)	IV (CurC)	IV
All sites	93.1%	88.3%	787.3%	76.4%	91.5%	80.6%	65.8%	86.0%	49.2%	17.7%	26.7%
	8081	7520	1399	642	1250	5079	1891	25,863	1483	4028	5511
Cecum	95.0%	88.9%	69.0%	78.1%	88.8%	72.9%	59.2%	83.6%	40.0%	13.5%	20.7%
	543	477	108	53	77	325	156	1739	119	356	475
Ascending colon	91.7%	87.2%	75.7%	72.5%	91.8%	81.8%	60.7%	84.6%	43.3%	16.2%	23.6%
	999	1333	257	65	128	777	278	3837	221	642	863
Transverse colon	92.0%	88.8%	77.5%	78.0%	89.4%	78.5%	63.3%	85.2%	49.8%	14.1%	25.2%
	732	773	184	65	71	413	163	2401	145	344	489
Descending colon	95.2%	86.8%	69.4%	78.1%	96.9%	81.9%	56.2%	85.1%	43.9%	21.0%	26.3%
	295	379	105	25	37	226	52	1119	49	187	236
Sigmoid colon	93.9%	89.4%	82.6%	80.1%	91.4%	84.1%	73.9%	88.2%	52.6%	19.0%	28.6%
	1970	1862	432	222	319	1281	364	6450	401	1091	1492
Rectosigmoid	93.1%	88.7%	85.8%	70.0%	93.7%	80.4%	72.3%	86.4%	56.0%	19.0%	29.5%
	909	897	177	87	166	707	247	3190	201	535	736
Upper rectum	93.7%	90.4%	73.9%	81.1%	95.5%	81.0%	59.1%	86.2%	46.6%	16.5%	24.7%
	903	839	116	38	165	721	240	3022	169	462	631
Lower rectum	92.2%	86.1%	81.7%	70.7%	88.5%	77.5%	68.1%	85.6%	52.0%	21.9%	31.3%
	1655	924	18	65	279	612	367	3920	159	380	539
Anal canal	91.0%	60.5%	50.0%	74.3%	87.5%	44.6%	47.9%	72.7%	33.3%	24.8%	31.3%
	75	36	2	22	8	17	24	184	19	31	50
Colon	93.3%	88.5%	77.6%	78.2%	91.3%	81.4%	65.4%	86.1%	47.9%	17.1%	25.7%
	4539	4824	1056	430	632	3022	1013	15,547	935	2620	3555
Rectum	92.7%	88.1%	75.0%	74.6%	91.0%	79.4%	64.5%	85.9%	49.3%	18.9%	27.8%
	2558	1763	134	103	444	1333	607	6942	328	842	1170

JSCCR colorectal cancer registry: patients in years 2008–2013. Staging was performed according to the rules set forth in the “Japanese Classification of Colorectal Carcinoma” (9th edition)

**Table 9:** Incidence of synchronous distant metastasis of colorectal cancer

	Liver	Lung	Peritoneum	Others
All sites	10.5%	3.6%	4.3%	3.9%
No. of patients (37,943)	3976	1353	1641	1495
Colon caner	11.2%	3.3%	5.5%	4.2%
No. of patients (25,621)	2581	754	1262	975
Rectal cancer and anal cancer	9.4%	4.0%	2.6%	3.3%
No. of patients (14,824)	1395	599	379	484

JSCCR colorectal cancer registry: patients in years 2008–2013

**Table 10:** Cumulative incidence of recurrence according to the site of recurrence (JSCCR colorectal cancer registry: patients in the year 2014)

pStage (no. of patients)	Recurrence rate (no. of patients with recurrence)	Cumulative incidence of recurrence according to the no. of years after surgery (cumulative no. of patients with recurrence)			Percentage of patients experiencing recurrence more than 5 years after surgery among all patients (no. of patients)
		3 years	4 years	5 years	
I (2141)	4.7% (100)	83.9% (100)	86.2% (73)	95.4% (83)	0.19% (4)
II (2516)	14.2% (356)	85.7% (294)	92.7% (318)	97.4% (334)	0.36% (9)
III (2558)	28.7% (735)	89.1% (735)	94.4% (631)	97.5% (690)	0.70% (18)
All (7215)	16.5% (1191)	87.7% (998)	93.2% (1061)	97.3% (1107)	0.43% (31)

JSCCR colorectal cancer registry (patients in the year 2014); 53 patients were excluded from analyses for cumulative incidence of recurrence because of unknown recurrence date

**Table 11:** Recurrence rate of pStage I colorectal cancer

pStage I	No. of patients	No. of patients with recurrence	Recurrence rate (%)	*p* value
Tumor location				
Colon	1281	38	3.0	< 0.0001
Rectum	860	62	7.2	
Depth of tumor invasion				
T1	1237	47	3.8	0.027
T2	904	53	5.9	
Tumor location and depth of tumor invasion				
Colon				
T1	798	24	3.0	0.91
T2	483	14	2.9	
Rectum				
T1	252	23	5.2	0.022
T2	315	39	9.3	

JSCCR colorectal cancer registry (patients in the year 2014)

**Table 12:** Recurrence rate according to the site of the first recurrence after curative resection of colorectal cancer and cumulative incidence of recurrence according to the number of years after surgery

Site of first recurrence (no. of patients)	Recurrence rate (no. of patients with recurrence (include overlaps))	Cumulative incidence of recurrence according to the no. of years after surgery (cumulative no. of patients with recurrence)			Percentage of patients experiencing recurrence more than 5 years after surgery among all patients (no. of patients)
		3 years	4 years	5 years	
Liver	5.7% (408)	95.2% (373)	98.0% (384)	99.5% (390)	0.03% (2)
Lung	5.0% (358)	87.2% (299)	93.0% (319)	97.1% (333)	0.14% (10)
Peritoneum	2.6% (186)	90.1% (163)	94.5% (171)	98.9% (179)	0.03% (2)
Local	1.6% (112)	78.5% (84)	87.9% (94)	94.4% (101)	0.08% (6)
Anastomotic	1.0% (72)	80.9% (55)	89.7% (61)	92.6% (63)	0.07% (5)
Other	4.0% (285)	83.6% (230)	91.3% (251)	95.6% (263)	0.17% (12)
All (7215)	16.5% (1191)	87.7% (998)	93.2% (1061)	97.3% (1107)	0.43% (31)

JSCCR colorectal cancer registry: (patients in the year 2014); 53 patients were excluded from analyses for cumulative incidence of recurrence because of unknown recurrence date

**Table 13:** Comparison of the recurrence rates between colon cancer and rectal cancer according to the site of the first recurrence

Site of recurrence	Colon cancer (4536 patients)	Rectal cancer (2679 patients)	*p* value
Liver	5.6% (252)	5.8% (156)	NS
Lung	3.6% (161)	7.4% (197)	< 0.0001
Peritoneum	3.2% (144)	1.6% (42)	< 0.0001
Local	0.7% (33)	3.0% (79)	< 0.0001
Other	3.3% (151)	5.0% (134)	0.0001
All	14.4% (651)	20.2% (540)	0.0001

JSCCR colorectal cancer registry: (patients in the year 2014); RS cancer was counted as rectal cancer

## Treatment guidelines for colorectal cancer

1. Treatment strategies for Stage 0 to Stage III colorectal cancer

1. Endoscopic treatment (Fig. 1)

Principles of indication

Applicable when there are minimal risk of lymph -node metastasis and the tumor’s size and location allow for en bloc resection.

Indication criteria for endoscopic resection

1. Intramucosal carcinoma or carcinoma with slight submucosal invasion
2. Size does not matter
3. Any macroscopic type
4. This method involves endoscopically resecting the diseased part of the large intestine and collecting the resected tissue.
5. Treatment methods include snare polypectomy (polypectomy) [Note 1]; endoscopic mucosal resection (EMR) [Note 2]; underwater EMR (UEMR) [24] [Note 2]; and endoscopic submucosal dissection (ESD) [3, 5] [Note 3]. There are also subtypes of ESD called precutting EMR [6] [Note 4] and hybrid ESD [6] [Note 5].
6. When determining the indication and treatment options for endoscopic therapy, information on tumor size, predicted wall invasion depth, macroscopic morphology, and LST subclassification, which is a growth and progression classification, is essential.

Comment

1. The purpose of endoscopic resection is both diagnostic and therapeutic. This method involves excisional biopsy, and a histological examination of the resected specimen is used to determine the effectiveness of the treatment and the necessity for additional surgical colorectal resection. (CQ1)
2. Diagnostic indicators for cT1 deeply invasive cancer include endoscopic findings, such as fullness, erosion, ulcer, fold convergence, deformity, and rigidity. These indicators are observed using white light endoscopy, image-enhanced endoscopy such as NBI/BLI [25], with magnifying endoscopic observation, and endoscopic ultrasound [26–28].
3. En bloc resection is essential for accurately diagnosing the resection margin and assessing the deepest cancer infiltration status.
4. The limit for reasonable en bloc resection using snare polypectomy or EMR is 20 mm in diameter [29].
5. Colorectal ESD is an endoscopic resection technique that allows for en bloc resection regardless of size and was approved for insurance coverage for early stage malignant tumors in April 2012. However, it is technically challenging and carries a high risk of complications such as perforation, so it is performed with careful consideration of the endoscopist's skill. Previously, insurance covered lesions measuring 20–50 mm in diameter. The revision of April 2018 eliminated the upper limit on tumor size, allowing insurance to now apply to early colorectal cancer with a maximum diameter of 20 mm or more. Insurance also applies to early colorectal cancer with fibrosis, even if the tumor is 20 mm or less in diameter.
6. The suction cap technique, or endoscopic mucosal resection with cap, has been reported to carry a high perforation risk when used for colonic lesions.
7. If the preoperative diagnosis confirms cancer accompanied by an adenoma (intramucosal cancer), piecemeal resection of the adenoma portion may be performed to avoid dividing the cancerous area; however, piecemeal resection generally carries a high rate of incomplete resection and local recurrence. In addition, multi-piecemeal resection, which complicates accurate histological assessment, should be avoided [29].
8. Regarding ESD, multiple single-center and multicenter prospective studies [30] have confirmed that for early stage cancers with intramucosal lesions of 20 mm or more up to T1a and no histological risk of metastasis, ESD is not only safe but also achieves long-term prognosis equivalent to surgery while offering advantages in patient quality of life. Furthermore, with advancements in ESD strategies and devices, colorectal ESD can now be performed more safely compared with earlier practice.
9. In terms of cost-effectiveness, ESD may be superior to piecemeal resection when post-treatment surveillance is considered [31]. Therefore, when the endoscopist possesses adequate skill, ESD is often preferred over piecemeal resection even for cancer accompanied by adenomas (intramucosal cancer).
10. After endoscopic resection, the resection site should be examined in detail to check for any residual lesions.
  Dye spray and magnified observation are useful for diagnosing residual lesions [28].
  If any intramucosal lesion remains, perform additional endoscopic resection.
11. Follow-up after endoscopic treatment [32]
  For pTis cancer treated with piecemeal resection and positive horizontal margins, colonoscopy should be performed approximately 6 months later to check for local recurrence. (CQ2)
  For pT1 cancer under follow-up, it is necessary to assess not only local recurrence but also lymph-node recurrence and distant metastasis. In addition to endoscopic examinations, follow-up should include imaging diagnostics, such as CT scans and tumor markers. (CQ2)
  Recurrence after endoscopic treatment of pT1 cancer often occurs within 3 years, but late recurrence is possible; thus, ongoing vigilance is required [33].

Note 1. Polypectomy: a method in which a snare is placed on the stalk of the lesion and cauterized with high-frequency current. It is primarily used for polypoid lesions. Recently, a technique called cold polypectomy, which does not use high-frequency current, has come to be used primarily for tumors smaller than 1 cm in size. However, this technique is not indicated for cancer, because it does not ensure sufficient deep margins. Therefore, qualitative diagnosis (differentiation between adenoma, SSL, and cancer) through magnified observation before endoscopic resection is important.

Note 2. EMR: a method in which saline or other liquid is injected locally into the submucosal layer to elevate the lesion, which is then cauterized and removed using a polypectomy technique. These include the snare method. It is primarily used for superficial tumors and large sessile lesions.

Underwater EMR (UEMR) [24] is a method that utilizes buoyancy by submerging the patient in water (with saline) instead of local injection.

Note 3. ESD: this is a procedure in which a sodium hyaluronate solution or similar is injected into the submucosal layer around the lesion to elevate the lesion, and then, ESD knife is used to make an incision around the lesion and dissect the submucosal layer to remove the tumor en bloc [3]. It is primarily indicated for large tumors that cannot be resected en bloc by EMR, especially early stage cancers.

Note 4. Precutting EMR: a technique in which a snare is performed without dissecting the submucosal layer at all after an incision around the lesion is made using an ESD knife or the tip of a snare.

Note 5. Hybrid ESD: a procedure in which an incision is made around the lesion using an ESD knife or the tip of a snare, followed by dissection of the submucosal layer and finally removing a lesion by snaring.

2) Surgical treatment (Fig. 2)

Principles of surgery

- The extent of lymph-node dissection to be performed during colorectal cancer surgery is determined based on the preoperative clinical findings and on the extent of lymph-node metastasis and depth of tumor invasion by the tumor observed intraoperatively.
- If lymph-node metastasis is recognized, or suspected based on the preoperative/intraoperative findings, D3 dissection is performed [34].
- If no lymph-node metastases are observed based on the preoperative/intraoperative diagnostic findings, lymph-node dissection is performed based on the depth of tumor invasion.
  1. Lymph-node dissection is unnecessary for pTis cancer (D0), because pTis cancer is not accompanied by lymph-node metastasis. However, D1 dissection may be performed when bowel resection is adopted.
  2. D2 dissection is necessary for pT1 cancer, because the incidence of lymph-node metastasis is approximately 10% and because approximately 2% of pT1 cancer is accompanied by intermediate lymph-node metastasis (Table 5).
  3. Although there is insufficient evidence describing the extent of lymph-node dissection for cT2 (MP) cancer, at least D2 dissection is necessary [35]. However, D3 dissection can be performed, because about 1% of cT2 (MP) cancer is accompanied by main lymph-node metastases (Table 5) and because preoperative diagnosis of depth of invasion is not very accurate.
- See CQ10 for indications for lateral lymph-node dissection (LLND) in rectal cancer.

Surgical treatment for rectal cancer

- The principle for radical surgery for rectal cancer is TME (total mesorectal excision) or TSME (tumor-specific mesorectal excision) [36–39].

[Indication criteria for sphincter-preserving surgery]

- Sphincter preserving surgery is indicated only when the following criteria are fulfilled: (i) resection with no oncologic remnant (both the distal and circumferential resection margins are negative = DM0, RM0) can be achieved, and (ii) the postoperative anal function can be maintained.

[Autonomic nerve-preserving surgery]

- Considering factors such as the degree of cancer progression and the presence or absence of macroscopic nerve invasion, preservation of autonomic nerves is attempted to preserve urinary and sexual functions as much as possible, provided that curability is unaffected.

[Indication criteria for LLND]

LLND is indicated when the lower border of the tumor is located distal to the peritoneal reflection and the tumor is cT3 or cT4 [40]. (CQ10)

Laparoscopic surgery

- The indications for laparoscopic surgery are determined by considering the surgeon’s experience and skills as well as tumor factors, such as the location and degree of progression of cancer, and patient factors, such as obesity and history of open abdominal surgery.
- Subgroup analysis of the JCOG0404 trial, a randomized-controlled trial conducted in Japan [41] showed a tendency for RS, cN2, obese cases, and T4 cases to have a worse prognosis following laparoscopic surgery. These factors should be considered when deciding indication for laparoscopic surgery.
- Laparoscopic surgery using a reduced number of ports, e.g., single-port surgery, has also been attempted; however, there have been no reports examining a large number of cases, and its efficacy and safety have not been fully established.

Robot-assisted surgery

- Robot-assisted surgery has been covered by insurance in Japan for rectal cancer since April 2018 and for colon cancer since April 2022. When introducing robot-assisted surgery for colorectal cancer, it is necessary to comply with the conditions for surgeons and facilities set by the Japan Society for Endoscopic Surgery and the other related societies. (CQ3)

Adjuvant therapy

- Adjuvant chemotherapy should be considered for pStage III colorectal cancer and for pStage II colorectal cancer with a high risk of recurrence after R0 resection. (CQ6, CQ7)
- For resectable rectal cancer, the efficacy and safety of perioperative treatment with chemotherapy, radiation therapy, or a combination of both are under investigation. (CQ11, CQ12)

Comment

[Optimal length of the bowel resection]

1. In D1, D2, and D3 dissections, the bowel resection margin is determined to ensure that the pericolic or perirectal lymph nodes, as defined in the "Japanese Classification of Colorectal, Appendiceal, and Anal Carcinoma [2]" are dissected.
2. The extent of the pericolic lymph nodes in colon cancer is defined by the relative position of the primary tumor and the feeding artery. However, metastasis of the pericolic lymph nodes more than 10 cm away from the tumor edge is rare. A multicenter prospective cohort study as a JSCCR research project investigated 2,996 resected cases of pStage I to III colon cancer and showed that only four cases (0.1%) had metastasis to pericolic lymph nodes located more than 10 cm away from the tumor edge. Furthermore, there was no difference in the distribution of metastatic lymph nodes depending on the location of the primary tumor and the feeding artery [42].
3. The extent of the perirectal lymph nodes in rectal cancer is defined as follows: the oral side is defined by the lowest plunge point of the sigmoid artery, while the anal side is defined by the distance from the tumor edge. For cStage 0–III cases, it is rare for intramural and/or mesorectal distal cancer spread to develop at a distance of 3 cm or more from the tumor edge in RS and Ra cancer, or 2 cm or more in Rb cancer [43–46]. Thus, the distal resection margin of the bowel and mesorectum should be determined to include this range.
4. It should be noted that pT4, pN2, M1 (Stage IV), and poorly differentiated rectal cancer cases are frequently accompanied by distal spread a long distance from the primary tumor edge [42, 44–46].

[TME/tumor-specific mesorectal excision (TSME)]

- Total mesorectal excision (TME) is a procedure that resects all the mesorectum just above the anal canal [36]. Tumor‑specific mesorectal excision (TSME) is a procedure for partially resecting the mesorectum according to the location of the tumor [39].

[Intersphincteric rectal resection (ISR)]

- ISR is a surgical procedure for treating lower rectal cancer located close to the anus to ensure the adequate distal margin by removing the internal anal sphincter.
- The indication criteria for ISR are as follows: (1) able to ensure the resection with clear circumferential surgical resection margin (no infiltration to the external anal sphincter or levator ani muscles); and (2) able to ensure the adequate distal surgical margin (in general, 2 cm or more for T2/T3 tumors and 1 cm or more for T1 tumors). ISR is not recommended for cases with poorly differentiated cancer and cases in which the anal sphincter tonus is decreased.
- In a JSCCR survey of 2,125 cases, the 5-year survival rate after ISR was equivalent to that for lower rectal cancer cases in the JSCCR colorectal cancer registry, but the 5-year local recurrence rate (including anastomotic recurrence) was relatively high at 11.5%. The local recurrence rate increased markedly with tumor invasion depth (4.2% for T1, 8.5% for T2, 18.1% for T3, and 36.0% for T4).
- As the extent of anal sphincter resection increases, postoperative defecatory dysfunction (e.g., fecal incontinence) becomes more severe. The incidence is particularly high in patients receiving preoperative radiation therapy, those with anastomotic leakage, and older adults [47–49].

[Transanal total mesorectal excision (TaTME)]

- TaTME is a surgical procedure in which a single-port endoscopic surgical device is used to perform retrograde dissection and mobilization of the mesorectum from the anal side.
- In patients with a narrow pelvis or obesity, where it is difficult to obtain a view from the abdominal cavity side, TaTME has advantages including the ease of confirming the dissected layer and the ability to determine the bowel resection line on the anal side under direct vision. However, there are some risks, such as intraoperative damage to the urethra, autonomic nerves, or intestines; contamination of the surgical field due to rupture of the purse-string suture that closes the anal side of the tumor; and local recurrence due to dissemination of tumor cells.
- A meta-analysis of non-randomized studies reported that no difference in the local recurrence rate between TaTME and conventional laparoscopic rectal surgery (3.5% for TaTME cases and 2.2% for laparoscopic surgery cases) [50]. Conversely, an analysis of registry data in Norway reported a high local recurrence rate (9.5%) after TaTME, leading TaTME to be not recommend in Norway [51]. TaTME is a technically demanding procedure that should be performed only by highly experienced surgeons in specialized centers (Table 6).

[Autonomic nerve-preserving surgery]

- The autonomic nervous system relevant to rectal cancer surgery consists of the lumbar splanchnic nerves*, superior hypogastric plexus*, hypogastric nerve*, pelvic splanchnic nerves#, and pelvic plexus. (*Sympathetic nerves, #Parasympathetic nerves)
- For urinary function, if one side of the pelvic nerve plexus is preserved [AN1–4], some function is maintained.
- The hypogastric nerve controls ejaculatory function, and the pelvic splanchnic nerve controls erectile function. To preserve male sexual function, complete conservation of the autonomic nervous system on both sides [AN4] is necessary.
- Whether or not LLND is performed, urinary and male sexual function may still be impaired even when the autonomic nervous system is fully preserved [52–54].

[Local excision for rectal cancer]

- Local excision is indicated for cTis cancer and cT1 cancer (slight invasion) located distal to the second Houston valve (peritoneal reflection).
- The purpose of local excision for rectal cancer is both diagnostic and therapeutic. This procedure is an excisional biopsy, and histological examination of the resected specimen determines the likelihood of complete cure and the need for additional treatment (colorectal resection with lymph-node dissection). The criteria for additional treatment are the same as those in “CQ1: What are the indication criteria for additional treatment for endoscopically resected pT1 colorectal cancer?”

[Aggregate data from the JSCCR colorectal cancer registry]

1. The incidence of lymph-node metastasis by site and depth of tumor invasion, the curative resection rate by stage, and the 5-year survival rate are presented in Tables 5, 7, and 8.
2. The 5-year survival rate after curative resection of pStage 0 to pStage III colorectal cancer by site was: 86.0% for all cases, 86.1% for colon cancer, 86.4% for RS cancer, and 85.9% for Ra/Rb cancer (cases in years 2008–2013).

2. Treatment strategies for Stage IV colorectal cancer (Fig. 3)

- Stage IV colorectal cancer is defined by synchronous distant metastases, including those to the liver, lung, peritoneum, ovaries, distant (extra-regional) lymph nodes, and other organs, such as bone, brain, or adrenal glands.
- If both the primary tumor and distant metastatic lesions are resectable, curative resection of the primary tumor should be performed, and resection of distant metastases should be considered.
- If distant metastatic lesions are resectable but the primary tumor is not, in principle, resection of both is not performed, and alternative treatments should be considered.
- If distant metastases are unresectable but the primary tumor is resectable, the indication for primary tumor resection should be evaluated based on clinical symptoms and potential impact on prognosis. (CQ5)

Comment

Synchronous hematogenous metastasis (liver, lung, brain metastasis, etc.)

1. The frequency of distant metastases is shown in Table 9.
2. Liver metastasis
  If the metastatic lesion is resectable, liver resection should be considered after confirming that primary tumor resection is curative.
  Regarding timing, simultaneous resection of the primary tumor and liver metastases is feasible and safe [55]. Alternatively, metachronous resection may be appropriate depending on surgical invasiveness and the patient’s condition. The impact on long-term prognosis remains unclear.
3. Lung metastasis
  If resectable, pulmonary metastasectomy may be considered after primary tumor resection.
  Metachronous resection is commonly performed.
4. Peritoneal metastasis
  Complete resection is strongly recommended for P1.
  For easily resectable P2, complete resection is recommended.
  The benefit of resecting P3 has not been established.
5. Ovarian metastasis (CQ22)
  If curative resection is possible, resection is recommended regardless of synchronous or metachronous presentation.
  If other unresectable distant metastases are present, chemotherapy is performed. However, if uncontrollable growth occurs despite systemic therapy and symptoms are present, palliative resection should be considered.
6. Distant (extra-regional) lymph-node metastasis
  Resection may be considered, although no comparative studies have clearly demonstrated a therapeutic benefit. Recent reports suggest that resection of para-aortic lymph-node metastases may achieve a cure or prolong survival in select patients [56–60].
7. Other distant metastases (to bones, brain, adrenal glands, etc.)
  Although case reports of resection exist, a definitive survival benefit has not been demonstrated.
8. Distant metastasis to multiple organs
  Metastasis to the liver and lungs is typical.
  If resection of the primary tumor and both liver and lung metastases are safe and feasible, it is recommended [61–65].
9. Adjuvant therapy after resection of distant metastases
  Due to the high risk of recurrence, adjuvant chemotherapy should be considered following curative resection of distant metastases. (CQ20, CQ21)

3. Treatment strategies for recurrent colorectal cancer (Fig. 4).
4. The goal of treatment for recurrent colorectal cancer is to improve prognosis and quality of life.
5. The main treatments are surgery, systemic therapy, and radiotherapy. Thermal ablation may be appropriate in experienced centers. (CQ18)
6. Select a treatment method based on the patient’s fully informed consent, considering factors, such as expected prognosis, complications, and post-treatment quality of life.
7. If recurrence is confined to one organ, actively consider removing the recurrent lesion if complete removal is possible through surgery.
8. If recurrence involves two or more organs, resection may be considered if each is resectable [61, 66]. This is effective for resectable liver and lung metastases, and resection is recommended.
9. There is an opinion that for resectable liver or lung metastases, a certain observation period should be allowed before resection in order to rule out occult metastases [68, 69].
10. For liver metastasectomy, laparoscopic surgery is recommended after assessing safety and indications. (CQ17)
11. In some cases, systemic therapy can render previously unresectable liver or lung metastases resectable, enabling curative surgery [69, 70].
12. The efficacy and safety of preoperative chemotherapy for resectable recurrent lesions are unclear, and its indication should be carefully considered. (CQ19)
13. Regarding adjuvant chemotherapy after resection of recurrent lesions, evidence of clear benefit is lacking, except for reports showing improved recurrence-free survival after resection of liver metastases. (CQ20)

Comment

[Lymph-node recurrence/peritoneal recurrence]

1. In general, lymph node or peritoneal recurrence after curative resection of the primary lesion should be considered part of systemic disease, and systemic therapy should be implemented with reference to the section on systemic therapy for unresectable advanced or recurrent colorectal cancer.
2. Resection may be performed only when the disease is controlled in cases of localized lymph node or peritoneal recurrence, but its effectiveness is unclear. Indications should be determined after carefully considering surgical tolerance and postoperative quality of life [56, 59, 71–73].
3. In some cases of localized lymph-node recurrence, radiotherapy has been effective [74–76].
4. Regarding ovarian metastasis, if radical resection is possible, it should be performed. Even if unresectable distant metastases are present, resection should be considered if there are subjective symptoms due to growth. (CQ22)

[Local recurrence of rectal cancer]

1. The extent of recurrence should be assessed using diagnostic imaging, and resection should be recommended only in cases in which complete resection is expected, taking into consideration the type of recurrence, symptoms, and physical findings. (CQ14)
2. There is much debate about the effectiveness of palliative resection for the purposes of prolonging life and alleviating symptoms, and the indications should be carefully considered [77].
3. In cases of unresectable local recurrence, if tumor shrinkage is expected to enable R0 resection, chemoradiotherapy is an option. On the other hand, in cases where R0 resection is clearly not possible, systemic therapy and, in cases where high-dose radiation is possible, local therapy (chemoradiotherapy or particle-beam radiation therapy) are options. (CQ15)

4. Treatment strategies for hematogenous metastasis

Representative organs for hematogenous metastasis of colorectal cancer include the liver, lungs, brain, and bones.

When resection becomes possible after systemic therapy (conversion therapy), resection is expected to result in a cure and improve prognosis compared with systemic therapy alone [78, 79].

1. Treatment strategies for liver metastases

Treatments for liver metastases include hepatic resection, systemic therapy, thermocoagulation, stereotactic body radiation therapy, and hepatic arterial infusion.

- Hepatectomy is recommended for curatively resectable liver metastases.
- Liver resection is generally a partial (non-systematic) resection.

Indication criteria for liver resection

1. The patient is capable of tolerating surgery
2. The primary tumor has been controlled or can be controlled.
3. The metastatic liver tumor can be completely resected.
4. There are no extrahepatic metastases, or they can be controlled.
5. The function of the remaining liver will be adequate.
6. In patients with unresectable liver metastases who can maintain an overall condition at a certain level (performance status (PS) 0 to PS 2), systemic therapy should be considered.
7. Thermal coagulation therapy includes microwave coagulation therapy (MCT) and radiofrequency ablation (RFA).
8. If the patient’s overall condition is poor (PS ≥ 3) or there are no effective drugs, best supportive care (BSC) should be provided.

Comment

[Liver resection]

1. Although conclusions about liver resection have not been drawn from cohort studies or randomized-controlled trials, favorable outcomes have been reported in selected cases that cannot be achieved with other treatments.
2. The 5-year survival rate after liver resection is 35–58% [80–83]. In a multicenter study in Japan, the 3-year survival rate of 585 patients who underwent liver resection was 52.8%, and the 5-year survival rate was 39.2% [84].
3. A comprehensive evaluation of the number, size, and location of metastatic lesions, as well as the predicted remnant liver volume, is essential to determine the feasibility of complete resection.
4. MRI has significantly higher sensitivity than CT for lesions smaller than 10 mm [85]. There is insufficient evidence to support the efficacy of FDG-PET in diagnosing and treating liver metastases [86]. Intraoperative contrast-enhanced ultrasound is also effective in detecting lesions that disappear after chemotherapy. (CQ16)
5. Resection should be performed to avoid cancer exposure at the margins [87–90].
  Regarding resection margin distance, some reports recommend ≥ 1 cm [91, 92], while others suggest that it is sufficient if there is no cancer exposure [93–96].
6. For synchronous liver metastases, resection of the primary tumor may precede liver resection after evaluating the curability of the primary tumor.
  No definitive conclusion exists regarding the timing of resection for synchronous liver metastases [97–99].
7. As prognosis is poor in patients with portal hilar lymph-node metastasis, some reports consider it an exclusion factor for hepatic resection [100–102].
  In a Japanese survey, the 5-year survival rate for patients who underwent hilar lymph-node dissection was 12.5% [84].
8. In cases of liver metastasis with resectable extrahepatic metastasis (mainly pulmonary), resection of both may achieve long-term survival or cure [61, 62, 66, 103, 104].
9. For repeat hepatectomy in recurrent liver disease, a 5-year survival rate of 21–52% has been reported. For recurrence in the remnant liver, resection should follow the same criteria as for initial hepatic resection [90, 105–113].
10. Although evidence is insufficient to confirm the benefit of adjuvant chemotherapy after liver resection, its use is recommended given the high recurrence rate. (CQ19, CQ20)
11. The efficacy of preoperative chemotherapy for resectable liver metastases remains unestablished. (CQ19, CQ20)

[Treatments other than resection]

1. Systemic therapy is performed in cases of unresectable liver metastasis.
2. Hepatic arterial infusion therapy is generally not recommended for patients with unresectable liver metastases. Thermal ablation therapy may be considered as an option when combined with medical therapy or surgical resection. (CQ18)
3. Treatment strategy for lung metastases
4. Treatment of lung metastases consists of pulmonary metastasectomy, systemic therapy, radiotherapy, and radiofrequency ablation.
5. Pulmonary metastasectomy is considered when the metastatic lung tumor is resectable.
6. Pulmonary metastasectomy includes systematic resection and partial (non-systematic) resection.

Indication criteria for pulmonary metastasectomy

1. The patient is capable of tolerating surgery.
2. The primary tumor has been controlled or can be controlled.
3. The metastatic lung tumor can be completely resected.
4. There are no extrapulmonary metastases or they can be controlled.
5. The function of the remaining lung will be adequate.
6. In patients with unresectable lung metastases whose general condition can be maintained at a certain level, systemic therapy should be considered.

If surgery is not tolerated, stereotactic body radiation therapy (SBRT) may be considered when the primary tumor and extrapulmonary metastases are controlled or controllable, and the number of pulmonary metastases is three or fewer [114]

- If the patient’s general condition is poor, provide appropriate BSC.

Comment

[Surgical resection of pulmonary metastases]

1. Although this conclusion was not derived from cohort studies or randomized-controlled trials, pulmonary metastasectomy has demonstrated favorable outcomes in appropriately selected cases that cannot be achieved with other treatments [103, 115–121].
2. The 5-year survival rate after pulmonary metastasectomy is 53–68% [122–125].
3. The significance of hilar and mediastinal lymph-node dissection has not been established [126–128].
4. In cases of resectable extrapulmonary metastases (mainly liver metastases), some reports suggest the effectiveness of pulmonary metastasectomy [62, 64, 103, 121, 129, 130].
5. Repeat lung resection for recurrent disease in the remaining lung has been reported to result in a 5-year survival rate of 20–52% [119–121, 124, 131, 132]. For recurrence in the remaining lung after primary pulmonary metastasectomy, the indication for lung resection should be carefully evaluated in accordance with the previously mentioned criteria for pulmonary metastasectomy.
6. There have been no large-scale studies to date evaluating adjuvant chemotherapy after surgery for pulmonary metastases. (CQ21)
7. Poor prognostic factors include the number of metastases, bilateral lung metastases, hilar and mediastinal lymph-node metastases, serum CEA levels before lung resection, primary lesion factors (T factors, N factors), and disease-free interval (DFI) [119–121, 123, 129, 130, 133–136]. These factors should be considered when deciding whether surgery and multidisciplinary treatment are appropriate [137].

[Radiotherapy]

Although there is insufficient evidence regarding SBRT (stereotactic body radiation therapy), there are domestic and international reports on the treatment of patients who tolerate surgery [138, 139].

[Ablation therapy]

Although definitive evidence supporting the effectiveness of RFA for lung metastases from colorectal cancer is lacking, it is reimbursed by insurance as a treatment option. The indications for RFA should be thoroughly evaluated by a cancer board, and strict adherence to the Japanese Society of Interventional Radiology’s Guidelines for the Appropriate Use of RFA for Expanded Indications is essential.

3) Treatment strategies for bone metastases
4) The treatment of bone metastases from colorectal cancer primarily involves systemic therapy; however, pain management and the prevention and treatment of fractures are essential for maintaining quality of life.
5) If urgent symptoms are absent, systemic therapy is prioritized, similar to the approach for other organ metastases.
6) In cases of pathological fracture, spinal cord compression, or impending risk, surgical intervention or radiotherapy may be considered.
7) The use of bone-modifying agents should be considered to reduce fracture risk.

Treatment of bone metastases

1. Local therapy: radiotherapy (conventional external radiotherapy, stereotactic body radiotherapy) and surgery (fixation, decompression, replacement)
2. Systemic therapy: standard systemic therapy, bone-modifying agents such as *RANKL* inhibitors and bisphosphonates, and analgesics (narcotic and non-narcotic)
3. Treatment strategies for brain metastases
4. Brain metastases are often identified in the context of systemic disease; however, because no highly effective systemic drugs are available, surgical resection or radiotherapy (stereotactic radiotherapy, whole -brain irradiation) should be considered for lesions in which a therapeutic benefit, such as symptom relief, preservation of neurological function, or prolonged survival, is anticipated.
5. The optimal treatment approach should be determined by assessing the patient’s overall condition, as well as the status of other metastatic lesions and brain metastases, including their size, location, and number.
6. Radiotherapy should be considered for cases that are not amenable to surgical resection.

[Surgical treatment]

Indication criteria for brain resection [140, 141]

1. The patient is capable of tolerating surgery.
2. The primary tumor has been controlled or can be controlled.
3. The patient has a life expectancy of at least several months.
4. Resection will not cause significant neurologic symptoms.
5. There are no metastases to other organs or they can be controlled.

[Radiotherapy]

- The purpose is to relieve symptoms, such as neurological deficits and increased intracranial pressure, and to prolong survival through local disease control.
- Whole-brain irradiation should be considered in cases of multiple brain metastases.
- If there are approximately 3–4 brain metastases measuring 3 cm or less, stereotactic radiotherapy should be considered.

Comment

[Surgical treatment]

1. Approximately 90% of brain metastasis cases also involve other organs, and prognosis remains poor even after resection [140].
2. Although the mean survival time after resection for solitary brain metastasis has been reported as 30–40 weeks, the effectiveness of surgical treatment has not been assessed in a sufficiently large cohort.
3. There is ongoing debate regarding the benefit of adding whole-brain irradiation after resection of brain metastases.

[Radiotherapy]

1. The symptom improvement rate is 60–80% [142, 143].
2. Stereotactic radiotherapy achieves local control in 80–90% of cases [144].
3. According to a systematic review, the median survival times (MSTs) after stereotactic radiotherapy, whole-brain irradiation, and BSC were 6.4 months (5.1–9.5 months), 4.4 months (2–9 months), and 1.8 months (0.5–2.5 months), respectively[145–147].
4. Prognostic factors include age, PS, the number of brain metastases, and the control status of extracranial lesions [148–150].
5. Currently, the addition of stereotactic radiotherapy should be considered in cases with an expected prognosis of several years. When stereotactic radiotherapy is administered, monotherapy is also regarded as a treatment option because of its quality-of-life benefit. However, as the intracranial recurrence rate is higher than with whole-brain irradiation, imaging follow-up at appropriate intervals is required.
6. Treatment strategies for other hematogenous metastases
7. For hematogenous metastases to the adrenal glands, skin, or spleen, resection should be considered if feasible. However, these metastases are often accompanied by metastases to other organs, and chemotherapy or radiotherapy is frequently used.
8. Systemic therapy
9. Systemic therapy includes adjuvant chemotherapy to prevent postoperative recurrence, as well as treatment for unresectable advanced or recurrent colorectal cancer, with the goals of prolonging survival and relieving symptoms.
10. Decisions on whether to initiate treatment and which regimen to use are made jointly by medical professionals and the patient through shared decision-making. This process involves providing relevant information and considering tumor-related factors (e.g., disease stage, histological type, primary tumor location, biomarkers), treatment-related factors (e.g., adverse events, quality of life (QOL), cost), and patient-related factors (e.g., age, comorbidities, tolerance to expected side effects, motivation for treatment) (shared decision-making [Note 1])

The following drugs are approved as standard treatments for colorectal cancer and are covered by the national health insurance system in Japan.

Cytotoxic anticancer drugs: Fluorouracil (5-FU), 5-FU + levofolinate calcium (l-LV), tegafur uracil (UFT), tegafur gimeracil oteracil potassium (S-1), UFT + calcium folinate (LV), capecitabine (CAPE), irinotecan hydrochloride hydrate (IRI), oxaliplatin (OX), trifluridine/tipiracil hydrochloride (FTD/TPI), etc.

Molecularly targeted agents: Bevacizumab (BEV), ramucirumab (RAM), aflibercept beta (AFL), cetuximab (CET), panitumumab (PANI), regorafenib hydrate (REG), encorafenib (ENCO), binimetinib (BINI), entrectinib (ENTR), larotrectinib (LARO), trastuzumab (TRA), pertuzumab (PER)

Immune checkpoint inhibitors: Pembrolizumab (Pembro), nivolumab (Nivo), ipilimumab (Ipi).

Note 1. Shared decision-making: A collaborative communication process in which medical professionals and patients work together to determine the most appropriate medical decision for the patient, forming the foundation of patient-centered care. Unlike traditional informed consent, shared decision-making involves greater patient engagement and places a stronger emphasis on the patient’s values, preferences, and life context.

1. Adjuvant chemotherapy
2. Adjuvant chemotherapy is systemic chemotherapy administered after curative (R0) resection, with the aim of preventing recurrence and improving patient prognosis.

General principles for the indications of adjuvant chemotherapy

1. Stage III colorectal cancer (colon or rectal) after R0 resection.
2. Adequate recovery from any postoperative complications.
3. PS of 0 or 1.
4. Preserved function of major organs.
5. Absence of serious complications, particularly bowel obstruction, diarrhea, or fever.
6. Adjuvant chemotherapy is recommended for patients with stage III colorectal cancer. (CQ6)
7. In patients with stage II colorectal cancer who have a high risk of recurrence, adjuvant chemotherapy should be considered. (CQ7).
8. For indications in elderly patients, refer to CQ8.
9. For adjuvant chemotherapy after resection of distant metastases, see CQ21 and CQ22.
10. For biomarker testing before perioperative systemic therapy, see CQ9.

Regimen

The adjuvant chemotherapy regimens listed below have demonstrated efficacy in clinical trials and are available as treatments covered by the national health insurance system in Japan.

**Table:** 

Oxaliplatin (OX) combination therapy	CAPOX FOLFOX
Fluoropyrimidine (FP) monotherapy	Capecitabine (Cape) 5-FU + l-LV UFT + LV S-1

Administration period

- The treatment period is generally 6 months.

Comment

1. Adjuvant chemotherapy should generally be initiated approximately 8 weeks after surgery.
2. For stage III colon cancer, OX-based combination therapy is recommended. This recommendation is supported by three randomized-controlled trials conducted in Europe and the United States, which confirmed a significant reduction in recurrence and improvement in prognosis compared with 5-FU + l-LV [151–153]. UFT + LV and Cape have shown non-inferiority to 5-FU + l-LV [154], and S-1 has demonstrated non-inferiority to UFT + LV [155]. However, the non-inferiority of S-1 to Cape has not been established [156]. (CQ6)
3. The optimal duration of OX in the adjuvant setting for stage III colon cancer was assessed in a pooled analysis of six randomized-controlled trials, including the Japanese ACHIEVE trial. Overall, the 3-month treatment group did not demonstrate statistical non-inferiority to the 6-month group (IDEA collaboration) [157]. However, in patients receiving CAPOX, similar recurrence prevention efficacy was observed, particularly in those at low risk. In the ACHIEVE trial, 3-year DFS was comparable between the 3- and 6-month groups [158], and the incidence of peripheral sensory neuropathy was significantly lower in the 3-month group [159]. (CQ6)
4. Although evidence for adjuvant chemotherapy following upfront surgery for rectal cancer is limited compared with colon cancer, the efficacy of cytotoxic agents is considered similar. Therefore, colon cancer trial evidence should be considered when making treatment decisions for rectal cancer. (CQ6)
5. In stage II colon cancer, a randomized-controlled trial in Japan found that 1 year of UFT monotherapy did not significantly reduce recurrence compared with surgery alone [160]. However, a prospective observational study suggested that UFT + LV may improve survival in high-risk stage II patients who meet one or more of the following criteria: T4 lesions, tumor perforation or penetration, poorly differentiated adenocarcinoma or mucinous carcinoma, or fewer than 12 dissected lymph nodes [161]. (CQ7)
6. In stage II/III colon cancer, adding irinotecan to 5-FU + l-LV has not demonstrated additional benefit in the adjuvant setting, and the combination is not recommended. Similarly, molecular targeted agents have not demonstrated efficacy in this setting, and their use is not recommended.
7. The role of adjuvant chemotherapy after curative resection of distant metastases remains controversial. In Japan, randomized-controlled trials have shown that UFT + LV or FOLFOX significantly reduced recurrence after curative resection of liver metastases compared with surgery alone [162, 163]. However, none of these regimens demonstrated an overall survival (OS) benefit. (CQ21, CQ22)
8. A meta-analysis of clinical trials in stage II/III colon cancer reported that *KRAS* and *BRAF* mutations (in the context of microsatellite-stable [MSS] tumors) are associated with high risk of recurrence, while MSI-H/dMMR status is associated with low recurrence risk [164, 165]. Furthermore, FP monotherapy is reported to be ineffective in MSI-H/dMMR cases and is not recommended [166, 167]. While testing for *RAS*, *BRAF* mutations, and mismatch repair deficiency (MSI testing or MMR-IHC) is covered by the national health insurance system, the clinical utility of using their status to determine indication or regimen selection remains unclear.
9. Systemic therapy for unresectable colorectal cancer
10. In the best supportive care (BSC) without any systemic therapy, the median survival time (MST) for patients with unresectable advanced or recurrent colorectal cancer is approximately 8 months [168]. Recent advances in systemic therapy have extended MST to over 30 months [169–172], although achieving a cure remains challenging.
11. The primary aim of systemic therapy is to prolong survival and alleviate symptoms by delaying tumor progression. In some cases of initially unresectable colorectal cancer, cure may be possible if systemic therapy is effective and all metastatic lesions are completely resected.
12. A meta-analysis of randomized-controlled trials involving individuals with PS 0–2 demonstrated that the systemic therapy group had significantly longer survival compared with the BSC group [168].
13. When evaluating systemic therapy, the first step is to determine its appropriateness (Fig. 5).
14. Patients suitable for intensive systemic therapy (Fit) are those with good general condition, preserved major organ function, no severe complications, and who are deemed able to tolerate combination therapy with OX, IRI, or molecular targeted drugs [the steps in the decision‑making process for first‑line treatment in unresectable colorectal cancer (Fig. 6)].
15. Patients with contraindications for intensive systemic therapy (Vulnerable) are those who, based on their general condition, major organ function, and comorbidities, are determined to be unable to tolerate combination therapy with OX, IRI, or molecular targeted drugs (Fig. 6).
16. Patients who are not suitable for systemic therapy (Frail) are those who have been determined to be unsuitable for systemic therapy due to deterioration of their general condition, major organ dysfunction, or severe comorbidities (Fig. 6).
17. For patients who are deemed suitable for systemic therapy, *RAS* (*KRAS/NRAS*) testing, *BRAF*V600E testing, and MSI/MMR-IHC testing will be performed before starting first-line treatment. In postoperative recurrence, if prior results are available, those will be applied in clinical practice (Fig. 7).
18. CET and PANI are indicated only for individuals with wild-type *RAS* (*KRAS/NRAS*).
19. Pembro is indicated only for patients with DNA mismatch repair deficiency (microsatellite instability-high [MSI-H]/mismatch repair deficient [dMMR]) or high tumor mutation burden (tumor mutation burden-high [TMB-H]). Nivo and Ipi are indicated only for patients with DNA mismatch repair deficiency (see Comment (2)).
20. ENCO and BINI are only indicated for patients with *BRAF*V600E mutation.
21. PER and TRA are only applicable to *HER2*-positive patients.
22. ENTR and LARO are only indicated for patients with *NTRK* fusion.

General principles underlying the indications of systemic therapy

1. The clinical or histopathological diagnosis has been confirmed as colorectal cancer.
2. The curative resection is not possible.
3. Patients are defined as “Fit” or “Vulnerable” depending on the general condition, the major organ function, and the presence or absence of serious comorbidities (refer to the package insert of each drug).

The steps in the decision‑making process for first‑line treatment in unresectable colorectal cancer (Fig. 6).

A regimen that has been shown to be useful in clinical trials and is available in Japan under health insurance.

First-line therapy (see CQ25):

- FOLFOX [173–175] + BEV [169, 170, 176]
- CAPOX [177] + BEV [176]
- SOX + BEV [169]
- FOLFIRI [175, 178] + BEV [170]
- S-1 + IRI + BEV [179]
- FOLFOX + CET/PANI [172, 180, 181] (see comment (1))
- FOLFIRI + CET/PANI [182, 183] (see comment (1))
- FOLFOXIRI [34] + BEV [171, 184, 185]
- Infusional 5-FU + *l*-LV [186, 187] + BEV [188, 189]
- Cape [190, 191] + BEV [192]
- UFT + LV [193–235] + BEV [196]
- S-1 + BEV [197]
- CET/PANI [198–200]
- Pembro (see comment (2) and CQ23 [201, 202].

Second-line therapy:

- Patients who are refractory or intolerant to a regimen containing OX
  FOLFIRI [175] + BEV [203, 204]
  CAPIRI + BEV [205]
  FOLFIRI + RAM [206, 207]
  FOLFIRI + AFL [208]
  S-1 + IRI [209] + BEV
  IRI [61] + BEV [210]
  FOLFIRI + CET/PANI [211, 212]
  CET/PANI + IRI [213, 214]
  Pembro [215] (see comment (2) and CQ23)
  Nivo [216] (see comment (2) and CQ (23)
  Ipi + Nivo [217] (see comment (2) and CQ23)
  ENCO + CET [218] (see comment (3))
  ENCO + BINI + CET [218] (see comment (3))
  ENTR [219] (see comment (6))
  LARO [219] (see comment (6))
- Patients who are refractory or intolerant to a regimen that includes IRI:
  FOLFOX [222] + BEV [203, 223]
  CAPOX [224] + BEV [203]
  SOX + BEV
  FOLFOX + CET/PANI
  Pembro [215] (see comment (2) and CQ23)
  Nivo [216] (see comment (2) and CQ23)
  Ipi + Nivo [217] (see comment (2) and CQ23)
  ENCO + CET [218] (see comment (3))
  ENCO + BINI + CET [218] (see comment (3))
  ENTR [220] (see comment (6))
  LARO [221] (see comment (6))
- Patients who are refractory or intolerant to a regimen that includes both OX and IRI:
  CET/PANI [225–229] + IRI [230, 231] (see CQ24)
  FTD/TPI + BEV [232–235] (see CQ24)
  FTD/TPI [236, 237] (see CQ24)
  REG [238]
  Pembro [216] (see comments (2), (5), and CQ23)
  Nivo [217] (see comment (2) and CQ23)
  Ipi + Nivo [218] (see comment (2) and CQ23)
  ENCO + CET [219] (see comment (3))
  ENCO + BINI + CET [219] (see comment (3))
  PER + TRA [239, 240] (see Comment (4))
  ENTR [220] (see comment (6))
  LARO [221] (see comment (6))

Third-line therapy and beyond:

- CET/PANI [225–229] + IRI (235,236) (see CQ24)
- FTD/TPI + BEV [232–235] (see CQ24)
- FTD/TPI [236, 237] (see CQ24)
- REG [238]
- Pembro [216, 241] (see comments (2), (5), and CQ23)
- Nivo [217] (see comment (2) and CQ23)
- Ipi + Nivo [218] (see comment (2) and CQ23)
- ENCO + CET [219] (see comment (3))
- ENCO + BINI + CET [219] (see comment (3))
- PER + TRA [239, 240] (see Comment (4))
- ENTR [220] (see comment (6))
- LARO [221] (see comment (6))

Comments

1. *RAS* (*KRAS/NRAS*) mutations occur in approximately 50% of patients with unresectable colorectal cancer, and anti-*EGFR* antibody drugs (CET, PANI) have been shown to be ineffective in patients with these mutations. Recent studies have also demonstrated that anti-*EGFR* antibody drugs are highly effective for patients with primary tumors located on the left side (descending colon, sigmoid colon, or rectum) during first-line therapy, but their benefit is limited in patients with right-sided tumors (cecum, ascending colon, or transverse colon) [172, 242]. Therefore, for patients eligible for systemic therapy, *RAS* (*KRAS/NRAS*) testing is recommended before initiating first-line treatment.
2. DNA mismatch repair (MMR) deficiency (MSI-H/dMMR) is identified in approximately 4% of unresectable colorectal cancers [243]. The efficacy and safety of Pembro in first-line treatment were evaluated in the randomized phase III KEYNOTE-177 trial [201, 202]. The efficacy and safety of Pembro, Nivo, and Ipi + Nivo in patients with prior systemic therapy were assessed in the non-randomized phase II KEYNOTE-164 and CheckMate-142 trials, respectively [216–218].

Therefore, for patients without contraindications to systemic therapy, MMR testing is recommended before initiating first-line treatment. MMR testing includes MSI testing and MMR–IHC testing; however, MMR–IHC testing is not currently a companion diagnostic for Nivo or Ipi + Nivo (see CQ23).

(3) In Japan, *BRAF*V600E mutation is detected in approximately 5% of patients with unresectable colorectal cancer, and those with this mutation have a poor response to systemic therapy and an extremely poor prognosis [244–246].

The efficacy and safety of ENCO + CET and ENCO + BINI + CET as second-line or third-line treatments for unresectable colorectal cancer with *BRAF*V600E mutations were evaluated in the BEACON randomized phase III clinical trial [219].

Therefore, *BRAF*V600E testing is recommended before first-line therapy in patients eligible for systemic therapy. Additionally, *BRAF*V600E testing is useful as an auxiliary diagnostic tool for Lynch syndrome, and it is recommended for patients with MMR deficiency who are suspected of having Lynch syndrome. For the basic requirements for *BRAF*V600E testing, refer to the “Japanese Society of Medical Oncology Clinical Guidelines: Molecular Testing for Colorectal Cancer Treatment, 5th Edition" (Japanese Society of Clinical Oncology).

(4) *HER2*-positive colorectal cancer accounts for 2–3% of patients with unresectable disease, and evidence suggests that anti-*EGFR* antibody drugs may have limited efficacy in this population [247–249].

The efficacy and safety of PER + TRA in previously treated patients with *HER2*-positive unresectable colorectal cancer were evaluated in the TRIUMPH non-randomized phase II clinical trial [239]. This study enrolled patients with *RAS* wild-type, *HER2*-positive colorectal cancer who were resistant or intolerant to fluoropyrimidine, OX, IRI, and anti-*EGFR* antibody drugs. However, given the limited efficacy of anti-EGFR antibody drugs in *HER2*-positive disease, PER + TRA therapy is recommended for patients without prior anti-EGFR antibody therapy.

*HER2* testing should be performed at an appropriate time before treatment initiation; however, considering the potential loss of tumor specimens due to multiple thin sections and the burden on laboratory personnel, it is reasonable to conduct MMR testing and *RAS/BRAF* testing concurrently before starting first-line therapy. Companion diagnostics for *HER2* testing, the “Ventana ultraView Pathway *HER2* (4B5)” (IHC) and “PathVision *HER2* DNA Probe Kit” (FISH), “Histra *HER2* FISH Kit” (FISH), and “Guadant360® CDx Cancer Gene Panel” are available. If *HER2* amplification is detected through a comprehensive cancer genome profiling test, an expert panel should determine *HER2* positivity and decide on PER+TRA.

(5) Most TMB-H cases of colorectal cancer have MMR deficiency, and non-MSI-H, TMB-H cases are estimated to account for approximately 6% [250].

The efficacy and safety of Pembro in patients with TMB-H solid tumors refractory or intolerant to standard treatment were evaluated in the KEYNOTE-158 non-randomized phase II clinical trial [241]. This study did not include colorectal cancer, and the efficacy of Pembro for TMB-H, non-MSI-H colorectal cancer remains unclear; however, it is reasonable to consider Pembro as a treatment option in TMB-H patients who have become refractory or intolerant to standard treatment (see CQ23). In Japan, the FoundationOne^®^ CDx Cancer Genomic Profile is reimbursed as a companion diagnostic for TMB-H.

(6) *NTRK* fusions are found in 0.21% (95% confidence interval [CI]: 0.16–0.28) of patients with unresectable colorectal cancer [251], and these mutations have been associated with poor prognosis [252].

The efficacy and safety of ENTR and LARO for solid tumors, including colorectal cancer with *NTRK* fusion, were evaluated using pooled datasets from three ongoing international phase I/II trials [220, 221]. In Japan, FoundationOne^®^ CDx, Cancer Genomic Profile (*ENTR*, *LARO*) and FoundationOne^®^ Liquid CDx Cancer Genomic Profile (*ENTR*) are reimbursed as companion diagnostics for *NTRK* fusion.

6. Radiotherapy
7. Radiotherapy encompasses adjuvant therapy aimed at preventing postoperative recurrence of rectal cancer, reducing tumor burden before surgery, and preserving anal function, as well as palliative radiotherapy aimed at alleviating symptoms and prolonging survival in unresectable advanced recurrent colorectal cancer.
8. Adjuvant radiotherapy
9. Adjuvant radiotherapy includes preoperative, intraoperative, and postoperative irradiation.
10. The goal of adjuvant radiotherapy is to improve local control of rectal cancer. Preoperative irradiation may further enhance anal sphincter preservation and resection rates. However, current evidence is insufficient to confirm a survival benefit from adjuvant radiotherapy.
11. Preoperative irradiation is indicated for “cT3 or deeper or cN positive,” postoperative irradiation for “pT3 or deeper or pN positive, surgical dissection surface positive (RM1), or unknown cancer infiltration to the surgical dissection surface (RMX),” and intraoperative irradiation for “surgical dissection surface positive (RM1) or unknown cancer infiltration to the surgical dissection surface (RMX)."

Comment

Preoperative irradiation (CQ11)

1. The advantages of preoperative irradiation include preventing dissemination during surgery, maintaining blood flow to the tumor, resulting in a high proportion of radiosensitive tumor cells, reducing digestive tract damage, because the small intestine is not fixed in the pelvic cavity, improving the R0 resection rate through tumor shrinkage, and enabling potential anal sphincter preservation (258). The disadvantages are the risk of overtreatment in early stage disease and an increased risk of postoperative complications.
2. Twelve randomized-controlled trials have evaluated preoperative irradiation (without chemotherapy) [250], and five demonstrated significantly better local control rates compared with surgery alone. However, only one showed an improvement in survival [254]. Furthermore, two meta-analyses reported improved local control with preoperative irradiation compared with surgery alone, and survival benefits in the subgroup receiving ≥ 30 Gy. However, the survival benefit remains controversial [255, 256].
3. Trials of short-course irradiation with 25 Gy in five fractions have been conducted mainly in Europe [254, 257]. Because the late effects of radiation are influenced by the size of a single fraction, long-term monitoring is needed, including evaluation of anal function and intestinal disorders. Regarding the benefit of adding short-course irradiation to TME, the Dutch CKVO 95–04 trial compared preoperative irradiation (25 Gy/5 fractions) + TME with TME alone. The 5- and 10-year local control rates were significantly higher in the combination group, but there was no difference in 5- and 10-year survival between the groups [149, 257, 258]. Furthermore, compared with surgery alone, preoperative radiotherapy was associated with a higher incidence of sexual dysfunction and intestinal disorders [259, 260].
4. Preoperative irradiation can shrink the primary tumor, allowing sphincter preservation. When the goal of preoperative irradiation is sphincter preservation, surgery should be performed after an appropriate interval for tumor shrinkage (6–8 weeks after completing radiotherapy) [261].
5. Four randomized-controlled trials in Europe and elsewhere have compared chemotherapy combined with preoperative irradiation. Results showed that preoperative chemoradiotherapy (CRT) was associated with a significantly higher incidence of acute adverse events than preoperative irradiation alone, but also achieved a significantly higher pCR rate. In two studies, excluding the short-course radiation study, the local recurrence rate was significantly lower in the preoperative CRT group, with no differences between groups in sphincter preservation or survival [262–265]. Randomized-controlled trials using 5-FU or capecitabine as combination chemotherapy demonstrated equivalent efficacy and safety [266, 267]. In trials assessing the addition of OX to fluoropyrimidines, three reported increased adverse events without improvements in pCR, local control, or survival [266, 268–270], while one found no difference in adverse events but significantly increased pCR and DFS [21]. The NCCN Guidelines recommend single-agent 5-FU or capecitabine as combination chemotherapy.
6. A study comparing preoperative with postoperative CRT showed that the preoperative CRT group experienced fewer serious adverse events and a significantly lower local recurrence rate. Therefore, preoperative CRT is recommended in the NCCN Guidelines.
7. Total neoadjuvant therapy (TNT), which incorporates systemic therapy into preoperative treatment, has been developed as an alternative to preoperative CRT, and results from several phase III trials have been reported. However, the optimal regimen has not been established, and a few prospective studies have been conducted in Japan; therefore, further evaluation of safety and efficacy is needed. (CQ12)
8. Non-operative management (NOM; active observation, watch-and-wait), in which surgery is omitted when a clinical complete response (cCR) is achieved after preoperative treatment, is not currently recommended. (CQ13)
9. Palliative radiotherapy
10. Pelvic lesions
11. The purpose is to relieve symptoms such as pain, bleeding, and bowel problems caused by pelvic tumors.

[Dose and fractionation]

- 1.8–2.0 Gy is delivered per fraction, for a total dose of 45 to 50 Gy.
- Depending on the patient’s overall condition and symptom severity, the dose per fraction may be increased to complete treatment over a shorter period.

b. Extrapelvic lesions

1. Bone metastasis

- The purpose is to relieve pain, prevent pathological fractures, and prevent or treat spinal cord compression.

[Dose and fractionation]

- For standard irradiation, dose schedules of 30 Gy/10 fractions, 20 Gy/5 fractions, or 8 Gy/1 fraction are commonly used. Pain response and pain relief rates are similar between single-fraction and multi-fraction irradiation; however, single-fraction treatment is associated with a higher re-irradiation rate when pain recurs [271]. In stereotactic radiotherapy, regimens such as 35 Gy/5 fractions or 24 Gy/2 fractions are commonly used.

(2) Liver metastasis

[Dose and fractionation]

- Various regimens are used in stereotactic body radiotherapy, but 40–60 Gy/5 fractions is commonly applied.

(3) Pulmonary metastasis

[Dose and fractionation]

- In SBRT for stage I non-small cell lung cancer, similar regimens are often applied to pulmonary metastases, with 40–55 Gy/3–5 fractions for peripheral lesions and 60 Gy/8 fractions or 75 Gy/25 fractions for central lesions.
- Radiotherapy may also be used to palliate symptoms such as bleeding or bronchial stenosis from lung metastases. Regimens, such as 20–24 Gy/4–5 fractions, 30 Gy/10 fractions, or 40–50 Gy/20–25 fractions, are often chosen, taking into account the patient’s overall condition and prognosis.

(4) Brain metastasis

[Dose and fractionation]

- The standard regimen for whole-brain irradiation is 30 Gy/10 fractions, but fractionation and total dose are individualized based on the patient’s overall condition and prognosis.
- In stereotactic radiotherapy, fractionation is determined by tumor diameter. For small brain metastases, 18–25 Gy/1 fraction is prescribed to the edge of the target volume. For brain metastases > 3 cm but up to 4–5 cm, fractionated stereotactic radiotherapy is indicated, with 27–35 Gy/3–5 fractions commonly used [272].

7. Palliative care

- Palliative care is a broad term for the management of various physical and psychological symptoms related to cancer.
- Palliative care spans from diagnosis to the end stage, with care tailored to the disease stage and presenting symptoms.
- In principle, cancer treatment should be undertaken under conditions where symptom relief is achieved [273], and palliative care should begin concurrently with surgical treatment and systemic therapy.
- Palliative care to improve the QOL of patients with end-stage colorectal cancer includes:

1. Pain relief
2. Surgical treatment
3. Systemic therapy
4. Radiotherapy
5. Counseling for psychiatric symptoms.

8. Surveillance after colorectal cancer surgery

1. Surveillance for recurrence after curative resection (curability A) of colorectal cancer

1. For pStage 0 (pTis cancer), regular endoscopic examinations should be conducted to monitor for recurrence at the resection margin and anastomotic site. Surveillance for recurrence in other organs is not required.
2. For pStage I to pStage III, surveillance is performed to detect recurrence in the liver, lungs, local regions, anastomotic sites, lymph nodes, and peritoneum. The following points should be emphasized:
  The surveillance period should extend for approximately 5 years after surgery, with shorter surveillance intervals during the first 3 years.
  It should be recognized that rectal cancer carries a higher incidence of lung metastasis and local recurrence compared with colon cancer.
  Figure 8 illustrates a recommended surveillance schedule after curative resection for pStage I to pStage III colorectal cancer, based on a comprehensive evaluation of recurrence sites, incidence, treatment outcomes, and current clinical practice in Japan (Fig. 8).

2) Surveillance after curative resection (curability B) of colorectal cancer and resection of recurrent lesions

1. The same surveillance strategy as that for Stage III disease is applied to pStage IV cases following R0 resection (curability B) and to patients undergoing resection of recurrent lesions. However, recurrence or repeat recurrence frequently occurs in organs previously affected by metastasis or recurrence, and the likelihood of recurrence beyond 5 years remains relatively high.
2. In cases assigned curability B due to R1 resection, a close surveillance schedule should be planned for organs in which residual cancer is suspected.

3) Surveillance for metachronous multiple cancers

- Perform colonoscopy for the purpose of surveillance for metachronous multiple colorectal cancers.

Comment

1. Purpose and target of surveillance
2. The goal is to improve prognosis by enabling early detection and treatment of recurrence [274]. Therefore, surveillance is carried out in patients who are considered eligible for treatment if recurrence is identified [275].
3. Recurrence rate, time of recurrence, and organ of recurrence
4. Figures 9 and 10 and Tables 10, 11, 12 and 13 present the results of an analysis from the 2014 JSCCR colorectal cancer registry. The study population included 7,215 individuals with colorectal cancer who underwent curative resection at 84 institutions participating in the registry, with a median follow-up of 5.5 years.
5. Recurrence timing and recurrence rate by organ (Fig. 10; Tables 10, 11, 12, 13).
6. Approximately 87% or more of recurrences occurred within 3 years after surgery, and more than 97% occurred within 5 years.
7. Recurrence beyond 5 years after surgery was observed in fewer than 0.5% of cases.
8. Compared with liver metastasis, pulmonary recurrence tended to present later.
9. Pulmonary, local, and anastomotic recurrences were more frequently seen in rectal cancer, whereas peritoneal recurrence was more common in colon cancer.
10. Characteristics by pStage (Fig. 9; Tables 10, 11)
11. pStage I
12. The recurrence rates for colon cancer and rectal cancer were 3.0% and 7.2%, respectively, with rectal cancer demonstrating a higher recurrence risk.
13. The recurrence rate was 3.8% for pT1 cancer and 5.9% for pT2 cancer.
14. Recurrence tended to occur slightly later in these patients than in pStage II and pStage III cases, with approximately 5% of recurrences arising after 5 years. However, these cases represented only about 0.2% of all patients.
15. pStage II, pStage III
16. The recurrence rates for pStage II and pStage III were 14.2% and 28.7%, respectively.
17. In both stages, more than 85% of recurrences were observed within 3 years after surgery.
18. The proportion of cases with recurrence beyond 5 years was approximately 0.4% for pStage II and 0.7% for pStage III.
19. Surveillance after curative resection (curability A) of colorectal cancer
20. The surveillance schedule on page 63 was developed with consideration of recurrence frequency by stage, the most common recurrence sites and timing, and current surveillance practices in Japan.
21. Although differences exist in the diagnostic modalities and schedules recommended across guidelines, current surveillance in Japan is generally more intensive compared with representative European and American guidelines (NCCN [276], ESMO [277], ASCO [275], and ASCRS [278]).

## Clinical questions

CQ1: What are the indications for additional treatment after endoscopically resected pT1 colorectal cancer? (Fig. 11).
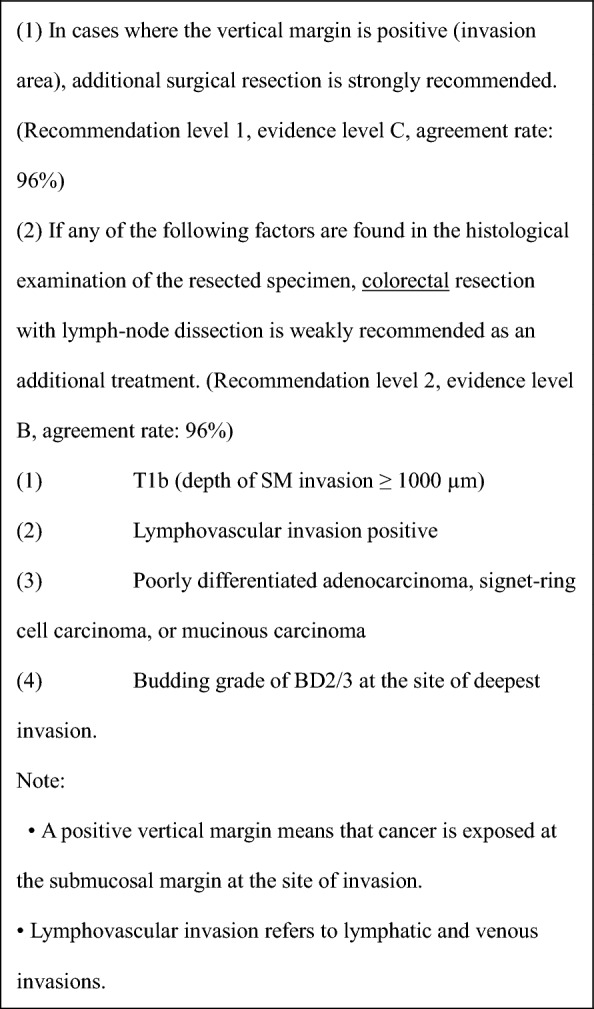


Risk factors for regional lymph-node metastasis in pT1 cancer include the depth of submucosal invasion [279], histological type (such as poorly differentiated adenocarcinoma, signet-ring cell carcinoma, or mucinous carcinoma [280]), the presence of poorly differentiated areas or mucinous component at the invasive front, budding G2/3, and lymphovascular invasion [280, 281]. A submucosal invasion depth of 1,000 μm has been established as the threshold for considering additional bowel resection and is identified as a risk factor for lymph-node metastasis in the Colorectal Cancer Treatment Guidelines for Physicians, 2005 Edition. However, even when the depth of submucosal invasion is 1,000 μm or greater, approximately 90% of patients do not develop lymph-node metastasis. Therefore, it is important to consider additional risk factors for lymph-node metastasis beyond submucosal invasion depth, including the location of the lesion, the physical and social background of each individual case, and the patient's preferences, to decide whether or not additional treatment is appropriate after thorough discussions among physicians, surgeons, and pathologists. In light of the above, additional bowel resection is “weakly recommended” for patients with even one risk factor for lymph-node metastasis in pT1 cancer.

It has been reported that the rate of lymph-node metastasis is relatively low if the only risk factor is submucosal invasion of 1,000 μm or more [282]. In a study of JSCCR, when the histological type of cancer was evaluated based on the worst differentiated component rather than the predominant histological type, the rate of lymph-node metastasis in patients with SM invasion of 1,000 μm or greater and negative for all other risk factors for lymph-node metastasis was 1.3% (95% CI 0–2.4%). According to the results of a recent study, the rate of lymph-node metastasis in patients with SM invasion of 2,000 μm or more was 11.0%, and the results of a nomogram-based lymph-node metastasis risk prediction model showed that patients with SM invasion of 2,000 μm or more had a higher risk of lymph-node metastasis [283]. Although further evidence is needed, additional bowel resection should be considered when submucosal invasion is 2,000 μm or greater, even if other risk factors for lymph-node metastasis are negative.

The 2009 edition added budding as a factor for which additional treatment should be considered, and project research on other histopathological factors is also underway. In addition, in cases of metastasis or recurrence, salvage surgery is often not possible, and there is a possibility of death from the cancer. This risk must be discussed with the surgeon.

CQ2: Is surveillance recommended after endoscopic resection of early stage colorectal cancer?
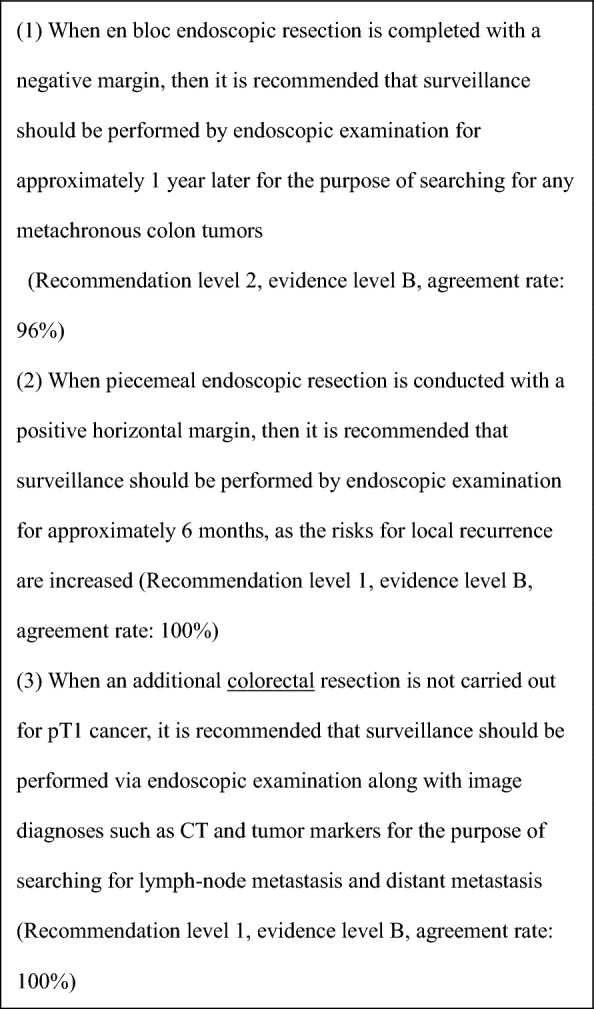


CQ3: Is robot-assisted surgery recommended for colorectal cancer?
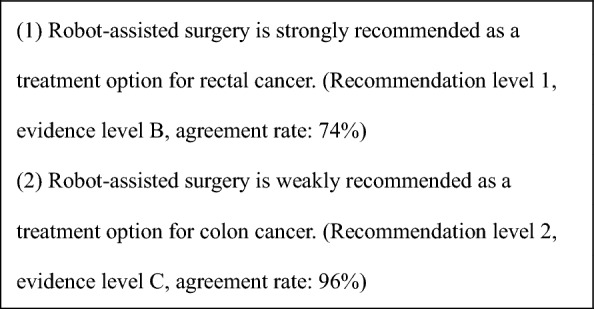


**<About robot-assisted surgery>**

Large-scale randomized-controlled trials and Cochrane reviews [284–295] have reported that while laparoscopic surgery takes longer operation time than open surgery, it offers superior short-term outcomes, including less blood loss, less postoperative pain, earlier recovery of postoperative intestinal peristalsis, and shorter hospital stays [284, 287–291]. In addition, the complication rates and long-term outcomes have been shown to be similar [286, 288, 292–295]. For these reasons, laparoscopic surgery has been accepted as one of the options for colorectal cancer surgery. On the other hand, the technical challenges of laparoscopic surgery have been pointed out, such as the limited range of motion caused by straight forceps and instability due to the surgeon's or assistant's hands tremor. Robot-assisted surgery has features not found in the conventional laparoscopic surgery, such as multi-jointed forceps with a wide range of motion, tremor reduction technology, a motion scale for precise operation, and operation under stable, high-resolution 3D images, and is expected to overcome the technical challenges of laparoscopic surgery. Robot-assisted surgery has been covered by insurance for rectal cancer since April 2018 and for colon cancer since April 2022.

**<Robot-assisted surgery for rectal cancer>**

Many cohort studies and meta-analyses have shown that robot-assisted surgery for rectal cancer reduces the rate of conversion to open surgery and the incidence of urogenital dysfunction compared to laparoscopic surgery [296–306]. Robot-assisted surgery has been reported to be superior or equivalent in terms of circumferential resection margin (CRM) positivity rate, complication rate, and length of hospital stay [296, 298, 300–302, 307]. However, it has been pointed out that the robot-assisted surgery is also associated with longer operative times and higher costs [308]. In addition, recurrence and survival rates have been reported to be equivalent to those of laparoscopic surgery [296, 302, 309, 310].

A large-scale cohort study from Japan using the National Clinical Database showed that in 20,220 cases of low anterior resection, robot-assisted surgery had a significantly lower conversion rate to open surgery than laparoscopic surgery (0.7 vs. 2.0%; *p* < 0.001). Additionally, robot-assisted surgery was shown to be superior regarding blood loss, in-hospital mortality, and length of postoperative stay, while laparoscopic surgery excelled in surgical time and readmission rate within 30 days after discharge [311].

In the ROLARR trial, the first large-scale randomized-controlled trial on robot-assisted surgery (237 patients in the robot-assisted group and 234 patients in the laparoscopic group), the conversion rate to open surgery, which was the primary endpoint, was 12.2% for laparoscopic surgery and 8.1% for robotic surgery, but superiority was not statistically proven [308].

In the REAL study (586 cases in the robot-assisted group and 585 cases in the laparoscopic group), a randomized-controlled trial focused on long-term oncological outcomes as the primary endpoint, a secondary analysis showed that robot-assisted surgery had a lower CRM positivity rate than laparoscopic surgery (4.0 vs. 7.2%; *p* = 0.023) and fewer postoperative complications (16.2 vs. 23.1%; *p* = 0.023) [312].

**<Robot-assisted surgery for colon cancer>**

Cohort studies and meta-analyses using large international databases have shown that robot-assisted surgery for colon cancer offers superior short-term outcomes compared to laparoscopic surgery, including a lower rate of conversion to open surgery, reduced incidence of complications, shorter hospital stays, and a greater number of lymph nodes harvested [313–319]. However, it has been pointed out that the procedure is time-consuming and costly [315]. Although there are limited reports on recurrence and survival rates, they have been reported to be equivalent to those of laparoscopic surgery [319–322].

A randomized-controlled trial of robot-assisted surgery for right-sided colon cancer reported no difference in short- or long-term outcomes compared with laparoscopic surgery; however, this was a small-scale study of 35 patients per group [321].

CQ4: Is stent treatment recommended for obstructive colorectal cancer?
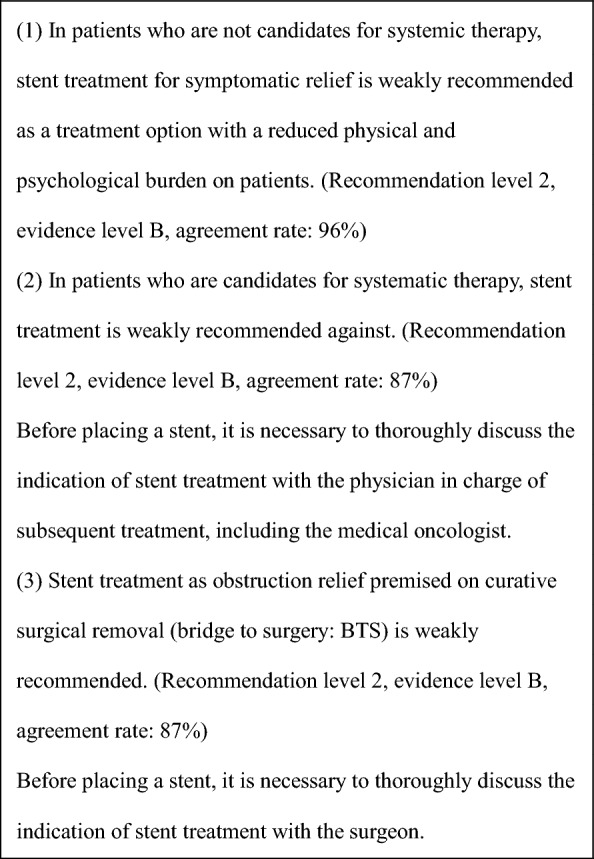


CQ5: Is primary tumor resection recommended for stage IV colorectal cancer patients with unresectable distant metastasis?
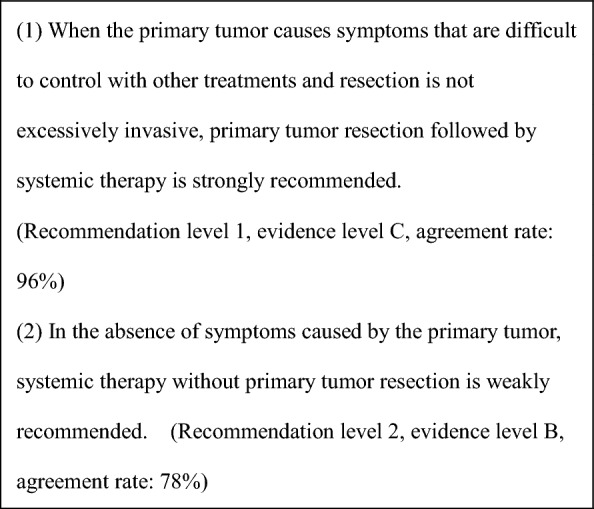


CQ6: Is adjuvant chemotherapy recommended for stage III colorectal cancer?
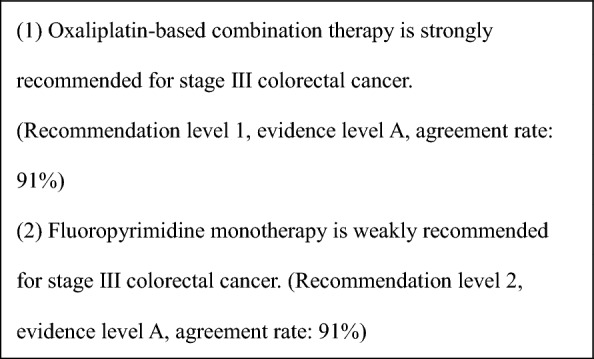


A pooled analysis of three randomized-controlled trials conducted in Europe and the United States involving Dukes’ B and Dukes’ C cancer showed that 5-FU + l-LV improved recurrence-free survival and OS compared to surgery alone [323]. Subsequently, in adjuvant chemotherapy for stage III colon cancer, it was demonstrated that a 6-month course of oxaliplatin (OX)-based combination therapy (CAPOX or FOLFOX) reduced the relative risk of recurrence or death by approximately 20% compared to a 6-month course of 5-FU + l-LV [151–153, 324, 325]. In Japan, the ACTS-CC 02 trial [326] for high-risk stage III disease failed to demonstrate the superiority of SOX therapy over UFT/LV, but the target sample size was not reached, and therefore, the findings should not be underestimated. Extrapolating results from Europe and the United States, CAPOX and FOLFOX regimens are also strongly recommended as effective treatment options in Japan.

**<Considerations regarding treatment period>**

In an investigation of adjuvant chemotherapy using OX-based combination therapy (FOLFOX, CAPOX) for stage III colon cancer, a pooled analysis of six randomized-controlled trials (TOSCA, SCOT, IDEA France, C80702, HORG, and ACHIEVE), including a domestic randomized-controlled trial (JFMC47-1202: ACHIEVE trial [327]), was conducted as part of the IDEA collaboration [157, 328]. Non-inferiority of the 3-month treatment group (test group) compared with the 6-month treatment group (control group) was not statistically demonstrated in either the primary endpoint of DFS or the secondary endpoint of OS (3-year DFS (*N* = 12,834) [15]: 74.6 vs. 75.5%, HR: 1.07, 95% CI 1.00–1.15; 5-year OS (*N* = 12,835) [328]: 82.4 vs. 82.8%, HR: 1.02, 95% CI 0.95–1.11). However, adverse events were less frequent in the 3-month treatment group, and the incidence of grade ≥ 2 sensory neuropathy was also significantly lower (6-month FOLFOX/CAPOX: 48%/45%; 3-month FOLFOX/CAPOX: 17%/14%). Additionally, a regimen-dependent interaction was observed, with the 6-month FOLFOX group showing superiority over the 3-month group, whereas the 3-month CAPOX group showed non-inferiority to the 6-month CAPOX group. Although not a pre-planned subgroup analysis, the non-inferiority of the 3-month CAPOX regimen was confirmed in patients with low recurrence risk (T1–3 and N1). A similar pattern was observed in the Japanese ACHIEVE trial [159, 327].

CQ7: Is adjuvant chemotherapy recommended for stage II colorectal cancer?
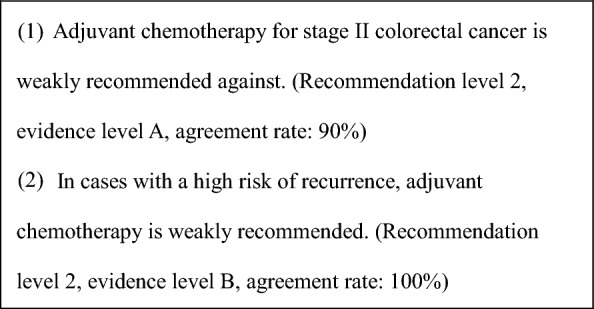


CQ8: Is adjuvant chemotherapy recommended for patients aged 80 years or older?
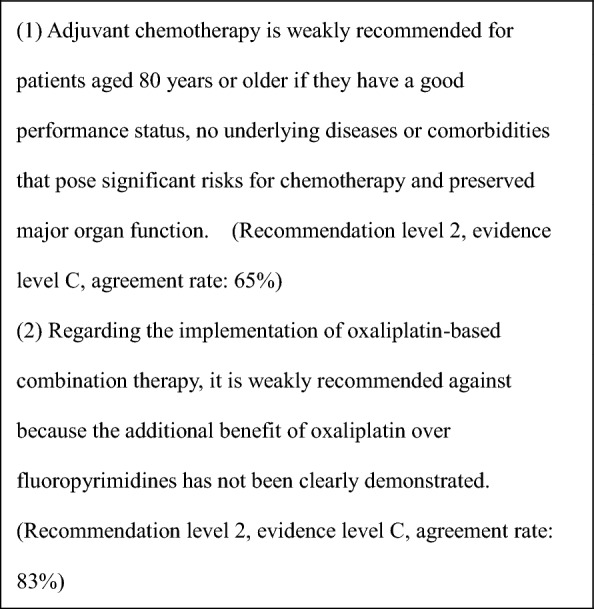


CQ9: Is biomarker testing recommended before perioperative systemic therapy?
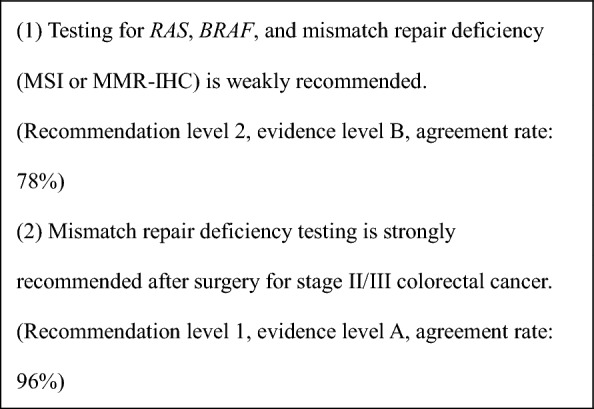


In current clinical practice in Japan, measurable biomarkers for colorectal cancer treatment include *RAS* gene mutations (up to 50% of cases), *BRAF* gene mutations (up to 7%), mismatch repair deficiency (MSI-H/dMMR: ~ 15%), and *HER2* gene amplification (up to 5%). However, as no prospective comparative studies using these biomarkers have been conducted in perioperative systemic therapy, meta-analyses and pooled analyses of retrospective studies were performed. There was insufficient information regarding perioperative systemic therapy and *HER2* gene amplification.

For 5-FU-based adjuvant chemotherapy in stage II/III colorectal cancer, meta-analyses of phase III trials demonstrated that *RAS* mutations were associated with worse DFS (HR: 1.36, 95% CI 1.15–1.61, *p* < 0.001) and OS (HR: 1.27, 95% CI 1.03–1.55, *p* = 0.03) compared with wild type. Similarly, *BRAF* mutations correlated with poor DFS (HR: 1.33, 95% CI 1.00–1.78, *p* = 0.05) and OS (HR: 1.49, 95% CI 1.31–1.70, *p* < 0.001) [329]. For patients with *BRAF* mutations, OS tended to be better with FOLFOX than with 5-FU/LV [330]. In MSI-H/dMMR patients, who generally have a favorable prognosis after surgery alone, adjuvant chemotherapy with 5-FU confers little benefit [331, 332]. In stage II cases, 5-FU monotherapy is often inferior to surgery alone and thus should not be administered [166, 333, 334]. Likewise, pooled ACCENT analyses showed that 5-FU alone did not improve survival in stage III MSI-H/dMMR colorectal cancer compared with surgery alone [335]. Conversely, when combined with oxaliplatin, DFS (HR: 0.47, 95% CI 0.27–0.82) and OS (HR: 0.52, 95% CI 0.28–0.93) were improved compared with 5-FU alone [335].

Regarding preoperative chemoradiotherapy for rectal cancer, no significant differences in treatment efficacy or survival have been observed between MSI-H/dMMR and MSS/pMMR groups [336, 337]. Immune checkpoint inhibitors are highly effective in MSI-H/dMMR locally advanced rectal cancer [338], and clinical trials are ongoing.

Although there is no disadvantage to biomarker testing before perioperative systemic therapy, testing for *RAS* and *BRAF* gene mutation testing may help predict prognosis and is weakly recommended. By contrast, mismatch repair deficiency (MSI/MMR-IHC) testing is valuable for postoperative treatment selection in stage II/III disease and is strongly recommended. In the future, emerging biomarkers such as ctDNA may be integrated.

CQ10: Is lateral lymph-node dissection (LLND) recommended for rectal cancer?
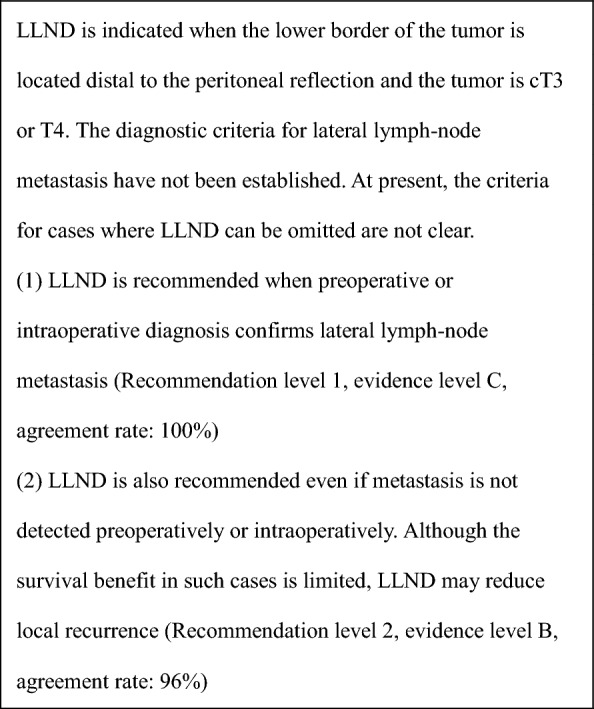


According to retrospective studies in Japan, 15–20% of low rectal cancer cases [40, 339–342] present with lateral lymph-node metastasis. In Europe and the United States, such metastasis is generally considered systemic disease and associated with poor prognosis; however, numerous reports indicate that when R0 resection is achieved, the 5-year survival rate is 45–55% [40, 340–342]. LLND is particularly effective in cases with few lymph-node metastases [343] and in cases limited to the internal iliac region [344]. A propensity score analysis of pT3 and T4 lower rectal cancers from the 1995–2004 JSCCR database showed superior 5-year OS for LLND cases compared with non-dissection cases (68.9 vs. 62.0%) [345]. A multicenter study further demonstrated improved prognosis with LLND in patients with mildly enlarged lateral lymph nodes on preoperative imaging [346]. Additionally, the benefit of lateral lymph-node dissection surpasses that of inferior mesenteric root lymph-node dissection, typically addressed in D3 procedures [342, 347]. Although retrospective studies are limited by inherent bias, consistent evidence suggests a survival benefit with LLND, underscoring its clinical significance. Even following preoperative chemoradiotherapy, patients with enlarged lateral lymph nodes before treatment show a high incidence of persistent metastasis; therefore, omission of LLND is not recommended [348–350].

Regarding the significance of LLND in cases without apparent lateral lymph-node metastasis, the JCOG0212 trial evaluated the non-inferiority of mesorectal excision (ME) alone compared with mesorectal excision plus LLND (ME + LLND) in patients with rectal cancer who did not have lateral lymph nodes with a short axis ≥ 10 mm on preoperative CT or MRI and whose tumor’s lower edge was located on the anal side of the peritoneal reflection, with recurrence-free survival as the primary endpoint. The HR for recurrence-free survival in the ME group compared with the ME + LLND group was 1.07 (90.9% CI 0.84–1.36). Because the upper limit of the non-inferiority margin was 1.34, non-inferiority of ME to ME + LLND was not statistically confirmed (P for non-inferiority = 0.0547) [351]. Local recurrence occurred significantly less frequently in the ME + LLND group (7.4%) than in the ME group (12.6%), although the recurrence-free survival curves of the two groups were very similar, and there were no significant differences in the secondary endpoints of OS or local recurrence-free survival. Conversely, recently published long-term follow-up results showed that the 7-year local recurrence-free survival rate (ME + LLND vs. ME: 82.9 vs. 78.9%) and 7-year lateral recurrence-free survival rate (ME + LLND vs. ME: 85.3 vs. 80.3%) were both superior in the ME + LLND group, and in subgroup analysis of cStage III, recurrence-free survival was significantly better in the ME + LLND group [352]. With respect to short-term surgical outcomes, the ME + LLND group had a prolonged operative time of approximately 100 min and increased blood loss of about 240 mL, and there was a trend toward more Grade 3–4 surgical complications in the ME + LLND group (21.7%) compared with the ME group (16.0%) [353]. Although no significant differences were observed in urinary or male sexual function [354, 355], the incidence of moderate-to-severe erectile dysfunction tended to be higher in the ME + LLND group than in the ME group [355].

In Europe and the United States, where LLND is considered a technically demanding procedure, it is recommended that this surgery not be performed when lateral lymph-node metastasis is judged to be negative [356]. However, based on findings from the JCOG0212 trial, from a local control perspective, LLND should not be uniformly excluded even when there are no enlarged lymph nodes in the lateral region. The potential advantages of LLND regarding local control and survival improvement should be recognized, and the appropriateness of the procedure should be determined by carefully balancing surgical risks against the possibility of postoperative functional impairment. The value of LLND in patients who have received preoperative chemoradiotherapy and are negative for lateral metastasis remains uncertain, and the invasiveness of combining local control therapies must be considered.

At present, the criteria for cases in which LLND may be omitted remain undefined. A randomized-controlled trial conducted in Japan indicated that preoperative irradiation yielded a therapeutic effect comparable to LLND; however, the number of evaluated cases was small [357], and reproducibility has not been established. Moreover, the accuracy of preoperative diagnosis of lateral lymph-node metastasis is inadequate, and the establishment of reliable diagnostic criteria remains an important challenge. A retrospective JSCCR study reported that MRI had superior diagnostic accuracy for lateral lymph-node metastasis when using a 5 mm short-axis cutoff compared with a 10 mm cutoff, although limitations remain when relying solely on lymph node size with conventional imaging [358]. A multicenter study currently underway by the JSCCR aims to establish diagnostic standards for lateral metastasis using high-resolution MRI, and it has been reported that combining long- and short-axis diameters allows extraction of lymph-node metastasis with a sensitivity exceeding 90% [359].

CQ11: Is preoperative treatment recommended for resectable rectal cancer?
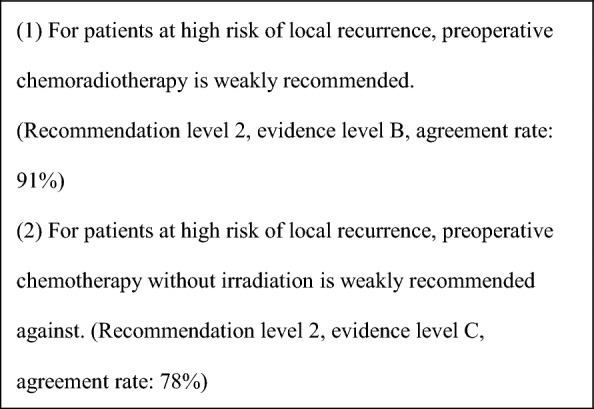


In Japan, TME (or TSME) + autonomic nerve-preserving LLND is the standard procedure for advanced lower rectal cancer, and good outcomes have been reported in terms of both survival and local recurrence rates [345, 351]. However, preoperative radiotherapy, which is standard in Europe and the United States, is not actively performed. In Japan, there is currently no clear evidence regarding the added benefit of preoperative radiotherapy in reducing local recurrence or its effectiveness as an alternative to LLND, and the standard treatment is upfront surgery.

The NIH Consensus Conference recommended a combination of chemotherapy and radiotherapy as adjuvant therapy for stage II or III rectal cancer [360]. Subsequently, a randomized-controlled trial comparing postoperative chemoradiotherapy with preoperative chemoradiotherapy was conducted and reported that the local control rate was higher in the preoperative chemotherapy group [361]. In Europe and the United States, preoperative radiotherapy is the standard for locally advanced rectal cancer; however, indications are stratified according to various risk factors (risk factors listed in the NCCN/ESMO guidelines: tumor location[low rectum], T3/4, positive lymph nodes, CRM involved, EMVI[ +]) and are not the same between Europe and the United States [362, 363]. Furthermore, preoperative radiotherapy increases the incidence of adverse events, such as intestinal disorders, bowel dysfunction, sexual dysfunction, and the development of secondary cancers [364–366]. Based on the above and referring to evidence from Europe and the United States, preoperative radiotherapy should be considered in patients who are predicted to be at high risk of local recurrence.

Outside of Japan, where preoperative chemoradiotherapy is the standard, emerging data suggest the effectiveness of preoperative chemotherapy without radiation, which aims to avoid the adverse events associated with radiation and improve survival rates by controlling distant metastasis, primarily in low-risk cases. The PROSPECT trial is a phase II/III trial in which low-risk patients with cT2N1, T3N0, or T3N1 (Stage IIA, IIIA, or IIIB) who are amenable to sphincter-preserving surgery were randomized to receive either preoperative chemoradiotherapy or preoperative chemotherapy (mFOLFOX6), with preoperative chemoradiotherapy selectively administered to non-responders in the preoperative chemotherapy group. The primary endpoint, the 5-year DFS rate, was 80.8% (95% CI 77.9–83.7) in the FOLFOX group and 78.6% (95% CI 75.4–81.8) in the chemoradiotherapy group, demonstrating non-inferiority of the FOLFOX group [367].

Regarding preoperative chemotherapy, the PROSPECT trial demonstrated the non-inferiority of LC-CRT compared to preoperative chemotherapy plus selective chemoradiotherapy in patients with a relatively low risk of local recurrence. However, there have been no comparative studies with upfront surgery, which is the standard treatment in Japan. Furthermore, in cases with a high risk of local recurrence, the efficacy of preoperative chemotherapy alone remains unclear, and thus, we weakly recommend against its use.

CQ12: Is total neoadjuvant therapy (TNT) recommended for rectal cancer?
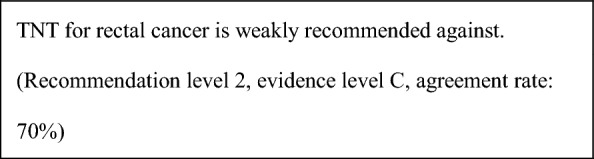


TNT is a treatment strategy that incorporates systemic therapy into preoperative treatment, replacing poorly tolerated adjuvant chemotherapy to compensate for the weaknesses of preoperative chemoradiotherapy (CRT), which improves local control but has not been shown to be effective in suppressing distant metastasis or improving prognosis.

Several randomized-controlled trials comparing TNT and CRT have been reported [368–373]. Regarding efficacy, the pathological complete response (pCR) rate was 28% in the RAPIDO study (vs. 14%) [371], and the PRODIGE23 trial reported a significant improvement in the pathological complete response (pCR) rate (28 vs. 12%) [370]. However, some reports have found no significant difference. On the other hand, there have been no reports of a significant improvement in the R0 resection rate in surgical cases. While no significant reduction in local recurrence has been observed, the latest report from the RAPIDO study indicated that the 5-year local recurrence rate was higher in the TNT group compared to the CRT group (12 vs. 8%), with most cases involving intraoperative perforation [373]. Both the RAPIDO [372] and PRODIGE23 [370] trials demonstrated significant reduction in distant metastasis recurrence rates (TNT vs. CRT): 23 vs. 30% at 5 years and 17 vs. 25% (3 years) at 3 years, respectively. They also reported improvements in disease-free survival (DFS): 72 vs. 66% at 5 years and 76 vs. 69% at 3 years. However, some studies have found no significant differences. To date, there have been no reports showing an improvement in overall survival (OS) after 5 years or more of follow-up.

The results of three meta-analyses comparing CRT with TNT, including these randomized trials, have shown that TNT consistently achieves high pCR rates; however, results regarding DFS and OS have been inconsistent, and this remains a subject of debate [374–376].

There is little data on TNT in Japan, and only the results of a single-arm phase II study from a single center reported to date. Konishi et al. evaluated the efficacy and safety of induction chemotherapy with FOLFOX plus bevacizumab followed by S-1/RT and reported a high pCR rate of 37.2% without an increase in postoperative complications, suggesting that this approach is both safe and feasible [377].

Results from large-scale clinical trials conducted thus far have demonstrated that TNT can increase the pCR rate and the likelihood of non-operative management (NOM), but its impact on OS remains unclear. Furthermore, there have been no reports comparing the difficulty of surgery or postoperative complication rates between TNT and upfront surgery, which is the standard treatment in Japan. Consequently, the safety profile of TNT has not been adequately established. Additional concerns include the potential for overtreatment, prolongation of treatment periods beyond six months, and increased medical costs.

Based on these considerations, we cautiously recommend against the routine use of TNT for rectal cancer under current circumstances. In particular, the uncritical adoption of TNT should be avoided at facilities lacking experience with preoperative radiotherapy. TNT should be conducted within well-designed clinical trial, and treatment outcomes in Japan should be clarified before considering treatment targets and optimal regimens.

CQ13: Is non-operative management (NOM) recommended for rectal cancer patients who have achieved cCR after preoperative treatment?
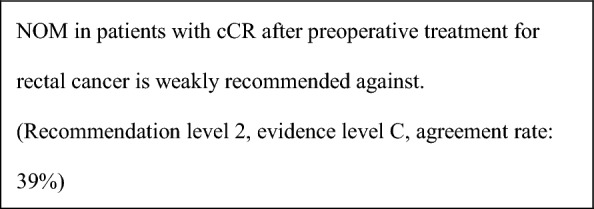


In Europe and the United States, neoadjuvant chemoradiotherapy (nCRT) is considered the standard treatment for cStage I to III rectal cancer, depending on the risk of recurrence. Furthermore, in 2018, the US NCCN guidelines added TNT, in which adjuvant therapy is administered before surgery, to the list of recommended treatment option [363]. If a clinical complete response (cCR) is achieved after preoperative treatment for such rectal cancer, non-operative management (NOM, also known as active observation or watch-and-wait therapy), in which wait-and-see treatment is performed without surgery, may be considered.

The OPRA trial [378] published in 2022 showed that organ preservation was possible in half of rectal cancer patients who underwent TNT and that consolidation chemotherapy after nCRT may be the optimal strategy to maximize pCR rates. Combined with the results of other phase II/III trials, approximately 10–20% of rectal cancer patients who received nCRT alone achieved a pCR [379, 380], whereas when a TNT regimen was added, the pCR rate could reach 20–60% [370, 378, 381].

Despite the positive results with NOM, one of the major uncertainties with NOM is the lack of long-term oncological outcomes [382]. Long-term active surveillance is essential for NOM. Currently, detailed evaluation using digital rectal examination, endoscopy, and MRI is considered the standard method, but this has not been thoroughly investigated. Tumor regrowth occurs in 25–30% of cCR patients (401], most of which occurs within the first year and generally plateaus after 2 years. Although local control is often achievable after regrowth, concerns remain regarding increased distant metastasis and worsened prognosis [383–385]. In addition, if salvage surgery is performed in patients with recurrence after NOM following preoperative treatment, an increase in adverse events and functional (urinary/sexual) disorders is expected compared to conventional surgery alone. Indeed, a recent multicenter study from the Netherlands found that quality of life and functional outcomes were worse when patients required surgery after NOM [386]. A Japanese study also reported that 41.7% of patients who underwent NOM after CRT experienced local uncontrollable tumor growth. It is important to note that nonoperative management without an appropriate surveillance protocol may worsen oncological outcomes [387].

In conclusion, in an environment where preoperative treatment is the standard of care, NOM may become a future treatment option for rectal cancer patients with cCR following preoperative treatment. However, the necessary shared clinical data between patients and providers are lacking before such strategies can be widely implemented. NOM involves replacing surgical resection with safe and active surveillance, and currently, there is no objective standardization to identify appropriate candidates for the NOM strategy without compromising oncological safety. This includes considerations of preoperative treatment regimens, definitions of cCR, and comprehensive surveillance methods, such as established diagnostic techniques for disease monitoring. Therefore, NOM should not be performed casually in patients with cStage I to III rectal cancer; instead, it should be conducted in prospective trials with established protocols and objective evaluation criteria.

CQ14: Is resection recommended for locally recurrent rectal cancer?
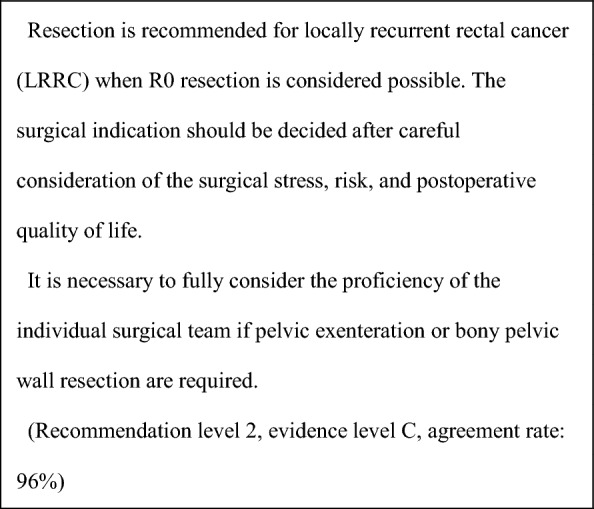


Surgery for locally recurrent rectal cancer (LRRC) often requires technically difficult procedures, such as total pelvic exenteration and bony pelvic wall resection. Resection should only be considered if it is determined that an R0 resection is possible, taking into consideration the proficiency of the individual surgical teams, including urologists and orthopedic surgeons. Regarding the high rate of postoperative complications and postoperative QOL, such as double stoma, it is essential to obtain sufficient informed consent from the patient and their family. In cases of R2 resection, both the prognosis and local control are clearly poor, and no improvement in quality of life can be expected. Therefore, resection is not recommended unless an R0 resection is anticipated [388–390].

Preoperative (re)irradiation has been reported to increase R0 resection rates and improve prognosis [391–394, 398], and it has also been reported that it can be performed relatively safely even in patients who have previously undergone radiotherapy by adjusting the irradiation method [398–401]. The usefulness of preoperative (re)irradiation for LRRC that is eligible for curative resection is being examined in a randomized trial being conducted in Japan in radiation-naïve patients (JCOG1801; NCT04288999) and in a randomized trial being conducted mainly in France in patients previously treated with radiation (GRECCAR15; NCT03879109). The results are currently awaited. A randomized-controlled trial (PelvEx-II; NCT04389086) is currently underway, mainly in the Netherlands, to verify the efficacy of adding preoperative chemotherapy to preoperative chemoradiotherapy for LRRC that is eligible for curative resection.

In addition, since April 2022, particle-beam radiation therapy—including both proton beam and carbon ion therapy, has been covered by the national health insurance system for unresectable pelvic recurrence of colorectal cancer. Particle-beam radiation therapy is capable of delivering high doses to localized areas and has been reported to produce better results than conventional radiation therapy [402–404]. Radiotherapy can be administered relatively safely even to patients with a history of radiotherapy [404]. Therefore, particle-beam radiation therapy may be an option in cases where R0 resection is difficult or where surgery is refused.

CQ15: Is radiotherapy recommended for unresectable locally recurrent rectal cancer without distant metastasis?
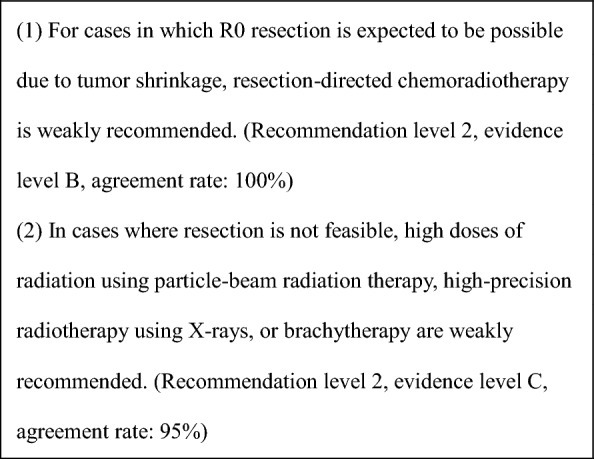


CQ16: Is resection of liver metastases that have disappeared on imaging as a result of successful systemic therapy recommended?
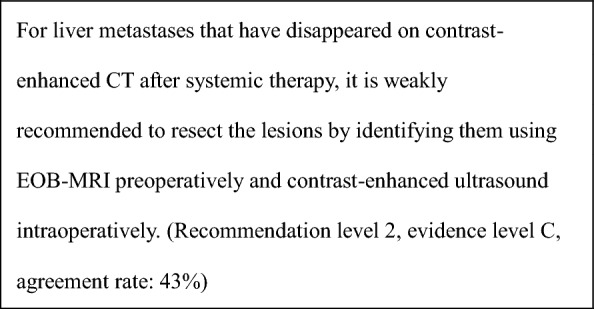


CQ17: Is minimally invasive surgery recommended for colorectal cancer liver metastases?
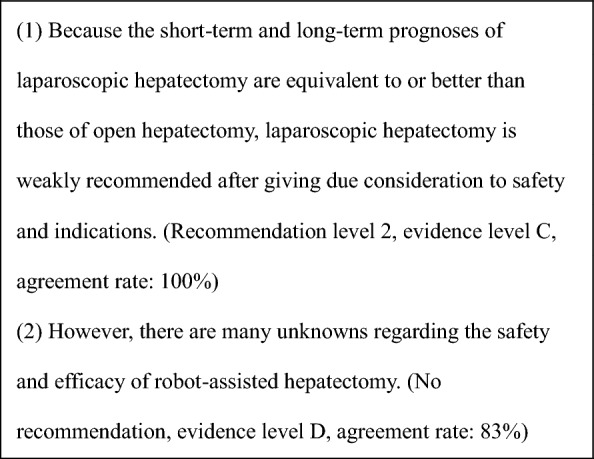


CQ18: Is thermal ablation therapy recommended for liver metastases?
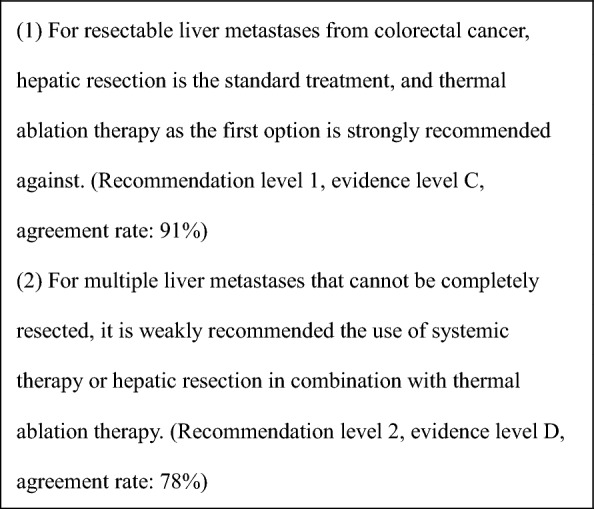


CQ19: Is preoperative chemotherapy recommended for resectable liver metastases?
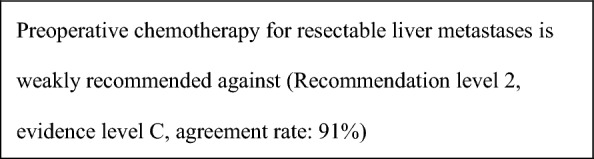


CQ20: Is adjuvant chemotherapy recommended after resection of liver metastases?
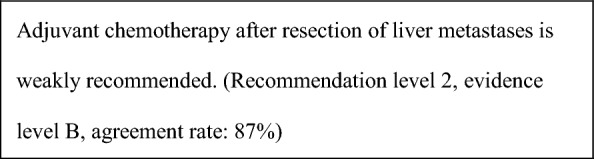


The most effective treatment for resectable liver metastases is definitive surgical resection. However, the recurrence rate after resection is high, ranging from approximately 45–70% [405–407], and improvements in treatment outcomes are necessary. Evidence for preoperative chemotherapy is currently insufficient, and its use is weakly discouraged. However, in real-world clinical practice, it may be administered to increase the likelihood of both technical and oncological resectability. In such cases, it is important that treatment decisions be made carefully through multidisciplinary team (MDT) discussions. For this reason, adjuvant chemotherapy has been considered to suppress recurrence and enhance prognosis.

A study by Hasegawa et al. [405] compared surgery alone with postoperative adjuvant chemotherapy using UFT + LV in patients who had undergone curative resection of liver metastases. The 3-year RFS was significantly better in the postoperative UFT + LV group (HR: 0.56, 95% CI 0.38–0.83), but there was no significant difference in OS (HR: 0.80, 95% CI 0.48–1.35), and this trend did not change even in the long-term follow-up of more than 7 years [408]. The JCOG0603 trial was a randomized-controlled trial comparing surgery alone with postoperative adjuvant chemotherapy using mFOLFOX6 therapy. The primary endpoint of DFS was significantly better in the postoperative adjuvant chemotherapy group (HR: 0.63, 95% CI 0.45–0.89), but there was no significant difference in OS (HR: 1.35, 95% CI 0.84–2.19) [407].

In conclusion, there are consistent results showing that adjuvant chemotherapy after resection of liver metastases is effective in suppressing recurrence but does not extend OS. It is believed that preventing or delaying recurrence can benefit patients if adjuvant therapy is administered appropriately, and postoperative adjuvant chemotherapy is weakly recommended. However, the decision to implement this treatment should be made on an individual basis, taking into account the current situation in which no evidence has been shown to extend survival time, the burden on patients due to hospital visits and adverse events associated with treatment, and the impact on repeat hepatectomy in the event of a recurrence due to liver damage.

CQ21: Is adjuvant chemotherapy recommended after resection of distant metastases other than hepatic metastases?
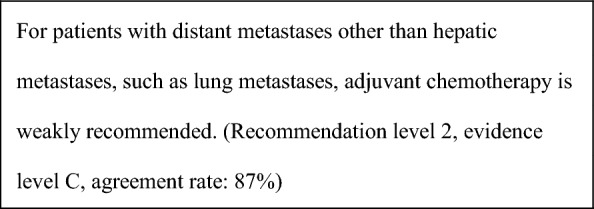


CQ22: Is ovarian resection recommended for ovarian metastasis from colorectal cancer?
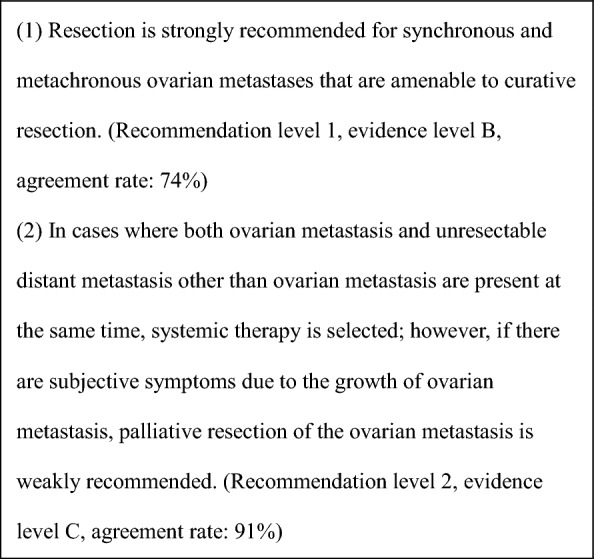


CQ23: Are immune checkpoint inhibitors recommended for unresectable colorectal cancer?
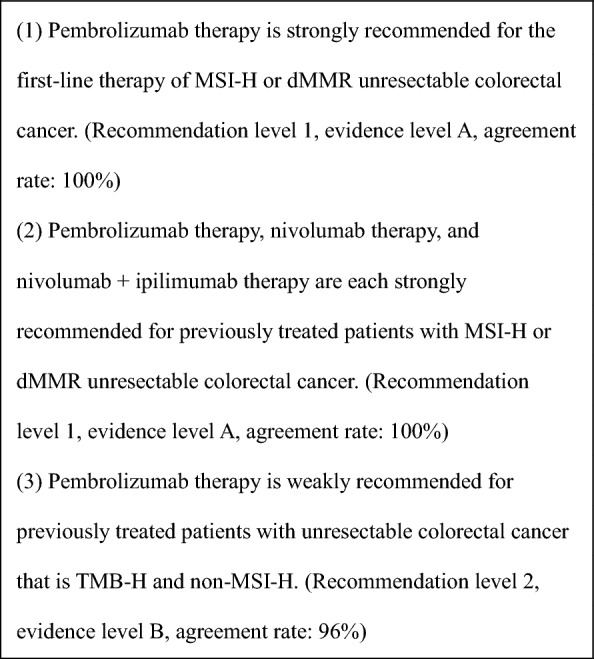


MSI-H/dMMR tumors account for approximately 4% among Japanese patients with unresectable colorectal cancer [411]. In the Phase III KEYNOTE-177 trial, pembrolizumab significantly improved PFS over chemotherapy [201] in patients with MSI-H or dMMR tumors, and OS also tended to be better (HR: 0.74, 95% CI 0.53–1.03) [202]. MSI or MMR-IHC testing should be performed prior to initiating treatment.

Separately, pembrolizumab is also approved for TMB-H (≥ 10 mut/Mb) solid tumors based on KEYNOTE-158, although colorectal cancer was not included. In non–MSI-H colorectal cancer (~ 6% of cases), the benefit remains uncertain. POLE/POLD1-mutated tumors may respond, but overall efficacy is limited. Therefore, pembrolizumab for TMB-H CRC should be considered cautiously, weighing TMB scores, mutation profiles, and alternatives like FTD/TPI ± BEV or regorafenib.

CQ24: Is late-line treatment recommended for unresectable colorectal cancer?
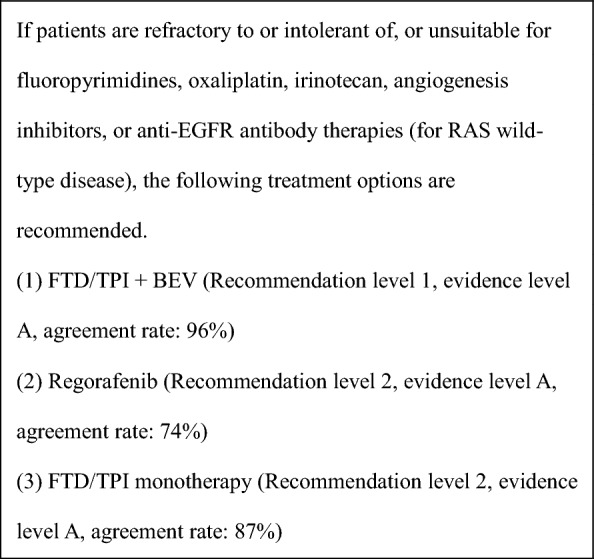


In patients with unresectable colorectal cancer who are refractory or intolerant to fluoropyrimidines, oxaliplatin, irinotecan, angiogenesis inhibitors, or anti-EGFR antibodies (for RAS wild-type disease), regorafenib and FTD/TPI have demonstrated survival benefits in placebo-controlled phase III trials, including Japanese patients. The CORRECT trial (regorafenib) and the RECOURSE trial (FTD/TPI) showed modest but significant improvements in OS compared with placebo. Moreover, FTD/TPI + BEV showed promising efficacy in Japanese phase I/II studies and was confirmed in the global phase III SUNLIGHT trial, which demonstrated a significant survival advantage over FTD/TPI alone (median OS: 10.8 vs. 7.5 months; HR: 0.61, *p* < 0.001). The clinical role of ctDNA-guided anti-EGFR rechallenge remains under investigation, and results from ongoing randomized trials are awaited to establish its role in later-line treatment. Fruquintinib, a VEGFR-1/2/3 tyrosine kinase inhibitor, has recently shown survival benefits in both the Chinese FRESCO trial and the global FRESCO-2 trial and is expected to become a new treatment option in this setting.

CQ25: Is maintenance therapy recommended after induction chemotherapy for unresectable colorectal cancer?
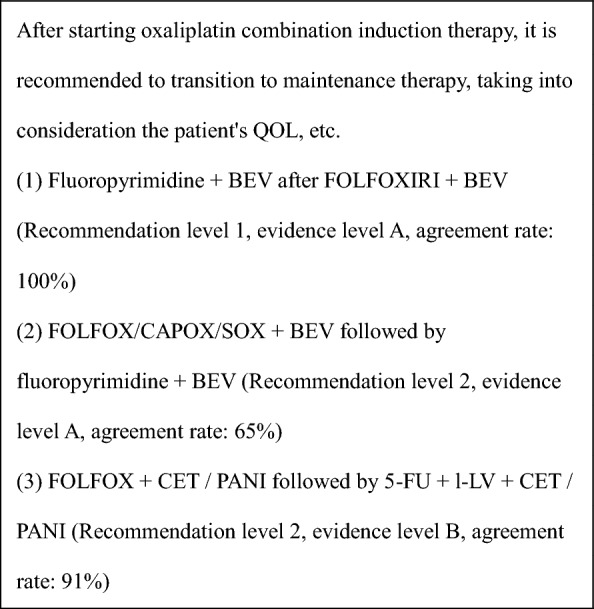


In unresectable colorectal cancer, intensive induction therapy (e.g., FOLFOXIRI plus BEV is initially used to achieve tumor shrinkage. Once maximal response is reached, treatment goals shift to disease control with reduced toxicity. Maintenance therapy with fluoropyrimidine plus bevacizumab after 8–12 cycles is commonly adopted to preserve quality of life.

Planned discontinuation of oxaliplatin (“Stop-and-Go”) is recommended after 12–16 weeks due to cumulative neurotoxicity. An IPD meta-analysis showed no survival difference between continuous and intermittent oxaliplatin use. Maintenance with fluoropyrimidine plus BEV prolongs PFS but not OS, while BEV monotherapy offers minimal benefit and is not recommended.

Temporary treatment holidays may also be considered, particularly when disease is stable, though careful patient selection and shared decision-making are essential.

Reintroduction of previously used agents may be effective if discontinued due to intolerance rather than resistance.

CQ26: Is comprehensive cancer genomic profiling recommended for unresectable colorectal cancer?
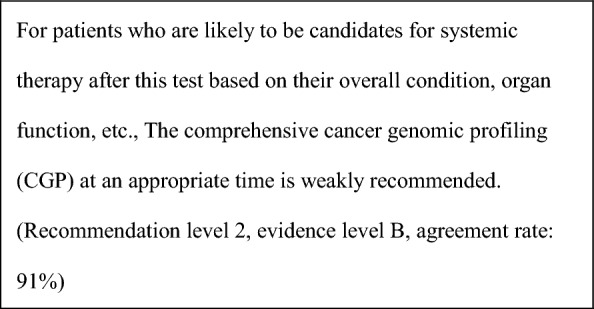


CGP identifies actionable genomic alterations to guide treatment. Besides tissue-based testing, plasma-based CGP is available for patients without accessible tumor samples. In colorectal cancer, targetable alterations including *NTRK*, *ALK*, and *ROS1* fusions, TMB-H and *HER2* amplification. Approved therapies include entrectinib/larotrectinib for *NTRK* fusions [412, 413], and pembrolizumab for TMB-H [414]. However, only 7% of patients receive CGP-guided therapy, and survival benefits remain unproven [415]. CGP testing is limited to designated hospitals and generally allowed only once per patient. Referral should be made at an appropriate time between initiation of first-line therapy and transition to later-line treatment.

CQ27: Is surveillance for multiple primary cancers (multiple colorectal cancers and other organ cancers) recommended after curative resection for colorectal cancer?
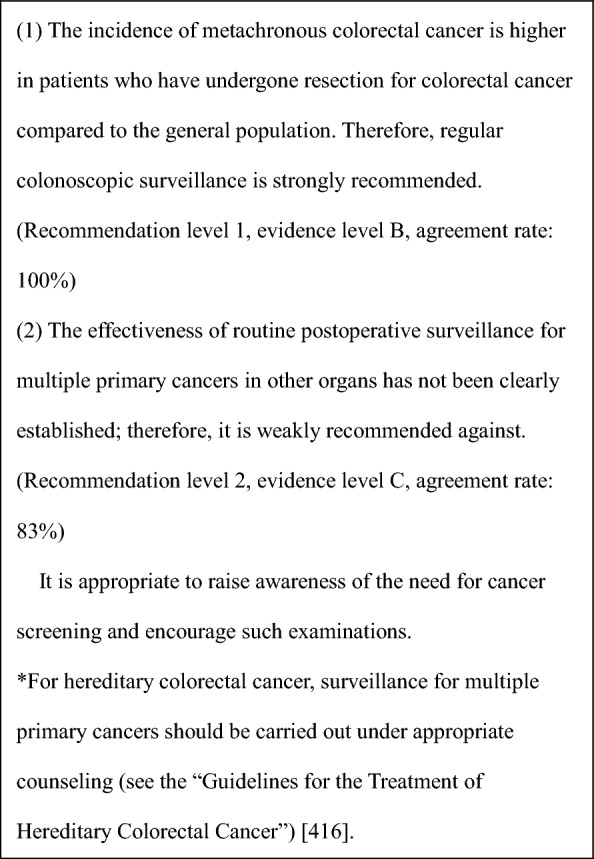


CQ28: Is chemoradiotherapy recommended for squamous cell carcinoma of the anal canal?
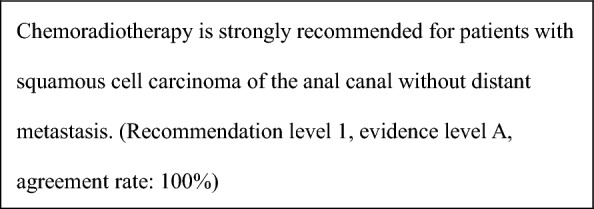


## Data Availability

The data used in this study are available from the corresponding author upon reasonable request.
